# Genetic Testing by Age at Onset in Parkinson Disease

**DOI:** 10.1001/jamaneurol.2026.1112

**Published:** 2026-05-11

**Authors:** Alexander Balck, Eva-Juliane Vollstedt, Ana Westenberger, Lara M. Lange, Joanne Trinh, Meike Kasten, Katja Lohmann, Norbert Brüggemann, Claudia Trenkwalder, Brit Mollenhauer, Roy Alcalay, Kamalini Ghosh Galvelis, James C. Beck, Peter Bauer, Christine Klein, Inke R. König, For the Global Parkinson’s Genetics Program (GP2), the ROPAD Study Group, and the PDGENEration Study

**Affiliations:** 1 Institute of Neurogenetics, University of Lübeck, Lübeck, Germany; 2 Section for Movement Disorders, Department of Neurology, University of Lübeck, Lübeck, Germany; 3 Laboratory of Neurogenetics, National Institute on Aging, National Institutes of Health, Bethesda, Maryland; 4 University of Lübeck, Lübeck, Germany; 5 Paracelsus-Elena-Klinik, Kassel, Germany; 6 Department of Neurology, University Medical Center Göttingen, Germany; 7 Gray Faculty of Medical and Health Sciences, Tel Aviv University, Tel Aviv, Israel; 8 Parkinson’s Foundation, New York, New York; 9 CENTOGENE, Rostock, Germany; 10 Institut für Medizinische Biometrie und Statistik, University of Lübeck, Lübeck, Germany; 11 Department of Neurosurgery, University Medical Center, Göttingen, Germany; 12 Neurological Institute, Tel Aviv Sourasky Medical Center, Tel Aviv, Israel; 13 Department of Neurology, Columbia University Irving Medical Center, New York, New York

## Abstract

This cohort study evaluates the association between age at onset and family history of Parkinson disease in patients who carry pathogenic variants.

Current recommendations for genetic testing in Parkinson disease (PD) prioritize patients with early age at onset (AAO; onset ≤50 years) and those with a positive family history (FH), despite limited data on the diagnostic accuracy of these criteria.^1,2^ Identifying causative PD genetic variants is increasingly relevant for counseling, prognostication, and eligibility for future gene-specific therapies. We evaluated how well AAO and FH identify carriers of pathogenic variants across large datasets.

## Methods

We analyzed 4 datasets comprising 25 063 individuals with PD, 6295 of whom carried pathogenic or likely pathogenic variants in *PRKN*, *PINK1*, *PARK7*, *LRRK2*, *SNCA*, *VPS35*, or *GBA1* (eMethods 1 and eFigure in Supplement 1). The Rostock International Parkinson’s Disease Study (ROPAD)^3^ and PD GENEration: Mapping the Future of Parkinson’s Disease (PD GENEration)^4^ studies are prospective clinical-genetic screening cohorts; the Movement Disorder Society Genetic Database (MDSGene),^5^ complemented by EPIPARK and DeNoPa, is a literature-based dataset of genetically confirmed PD plus control individuals with idiopathic PD; the Global Parkinson’s Genetics Program (GP2)^6^ is a global genotyping/sequencing resource (eMethods 1 in Supplement 1). In each dataset, we classified participants as having genetic PD or idiopathic PD and evaluated AAO and FH (any affected relative or first-degree relatives, depending on cohort) as predictors. Pathogenic and likely pathogenic variants were defined according to contemporary American College of Medical Genetics and Genomics criteria in the above genes, and all remaining participants were classified as having idiopathic PD. We used logistic regression models, including AAO and FH, to derive receiver operating characteristic curves, areas under the curve (AUCs), sensitivity, and specificity in all cohorts and to estimate positive predictive values (PPVs) in cohorts where the genetic PD prevalence could be reliably determined (ROPAD and PD GENEration; eMethods 2 in Supplement 1).

Across datasets, inclusion and exclusion criteria varied, and in MDSGene and GP2, many patients lacked complete AAO information, potentially introducing selection bias and contributing to variation in performance estimates across cohorts (eMethods 2 in Supplement 1).

## Results

**Figure 1.**
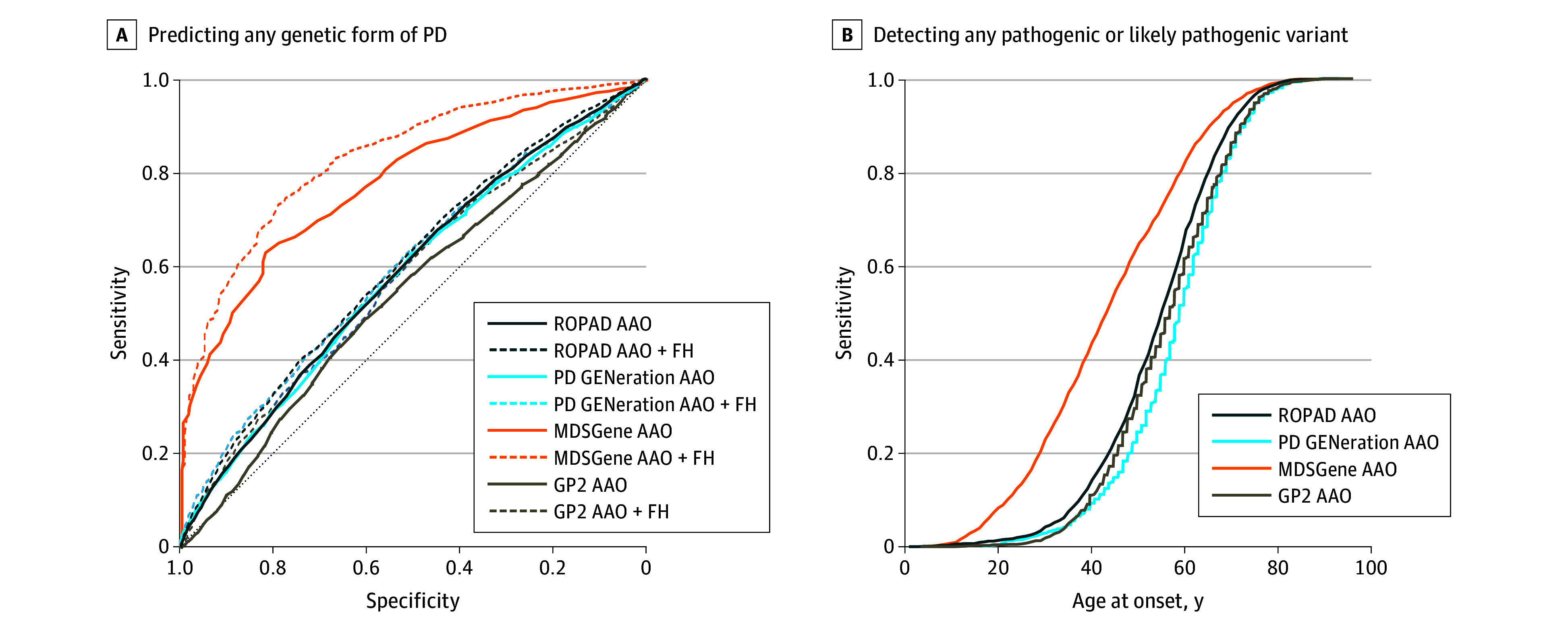
Performance Curves Showing Age-at-Onset (AAO) Criteria for Genetic Testing in Parkinson Disease A, Area under the receiver operating characteristic curve (ROC) for predicting any genetic form of Parkinson disease using AAO alone (solid lines) or in combination with positive family history (FH) (dashed lines) across 4 datasets (Global Parkinson’s Genetics Program [GP2], Movement Disorder Society Genetic Database [MDSGene], PD GENEration: Mapping the Future of Parkinson’s Disease [PD GENEration], and Rostock International Parkinson’s Disease Study [ROPAD]). B, Sensitivity curves for detecting any pathogenic or likely pathogenic variant across the 4 datasets as a function of AAO; at AAO 50 years, sensitivity was 32%, 23%, 30%, and 63% in ROPAD, PD GENEration, GP2, and MDSGene, respectively.

AAO alone showed modest discrimination for any genetic PD vs idiopathic PD (AUC, 0.59; 95% CI, 0.57-0.60 in ROPAD and 0.58; 95% CI, 0.56-0.60 in PD GENEration), higher in the literature-based MDSGene dataset (AUC, 0.78; 95% CI, 0.75-0.80), and low in GP2 (AUC, 0.54; 95% CI, 0.52-0.56) (Figure 1A). Using AAO 50 years and younger as the testing criterion identified 32% of variant carriers in ROPAD and 23% in PD GENEration (Figure 1B); thus, 68% to 77% of carriers would not meet current early-onset testing recommendations in these cohorts. Sensitivity was higher in MDSGene (63%), reflecting enrichment of this literature-based dataset with early-onset and recessive PD patients compared with unselected cohorts (Figure 1B). This pattern raises the possibility of a self-reinforcing bias, in which early publication of predominantly patients with early onset informed testing criteria that now preferentially identify the same subset. Adding FH increased AUC to 0.60 in both ROPAD and PD GENEration, indicating limited incremental value of FH in unselected patients.

When restricted to recessive early-onset genes (*PRKN*, *PINK1*, *PARK7*), AAO distinguished genetic from idiopathic PD with AUCs of 0.90 to 0.95 across datasets, showing that current criteria perform well for these rare forms. In contrast, AAO had limited discriminative ability for *LRRK2*- and *GBA1*-related PD (AUCs generally around 0.5-0.6); AAO 50 and younger identified 21% and 10% of *LRRK2* carriers and 30% and 21% of *GBA1* carriers in ROPAD and PD GENEration, respectively.

## Discussion

**Figure 2.**
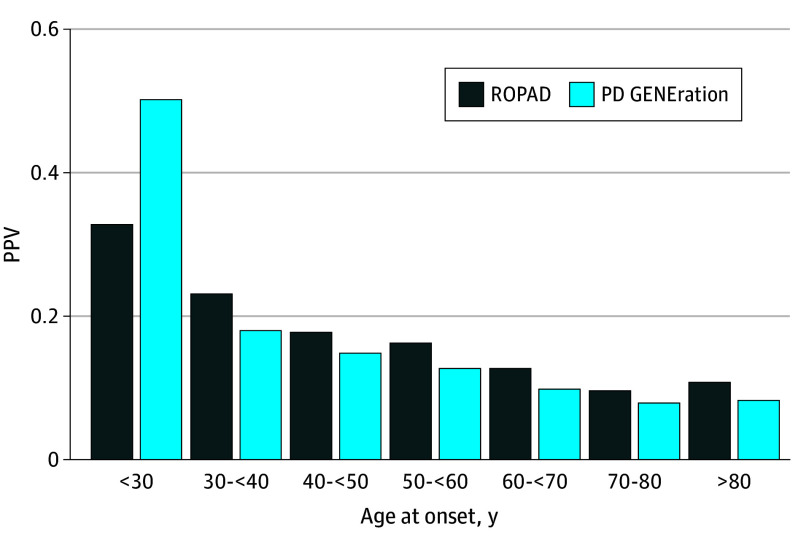
Bar Graph Showing Age Bracket Performance of Age-at-Onset (AAO) Criteria for Genetic Testing in Parkinson Disease (PD) Positive predictive values (PPVs) for any genetic PD across AAO categories in Rostock International Parkinson’s Disease Study (ROPAD) and PD GENEration: Mapping the Future of Parkinson’s Disease (PD GENEration); PPVs were highest at young ages and remained approximately 0.10 for AAO 60-70 years and 0.08 for 70-80 years, indicating substantial diagnostic yield even at older onset.

In practical terms, many patients with *LRRK2*- or *GBA1*-associated PD who first present with motor symptoms in their 60s or 70s would therefore not be offered testing under current criteria, even though they carry variants that are the focus of ongoing gene-targeted trials. Expanding testing beyond the 50-year threshold would directly affect the identification of such patients and their ability to participate in genotype-driven interventional studies. Despite lower variant prevalence at older ages, in PD GENEration, the PPV for any genetic PD was approximately 0.10 for AAO 60 to 70 years and 0.08 for 70 to 80 years (Figure 2). Thus, among patients with onset after age 60 years, roughly 1 in 10 to 1 in 12 tested individuals carried a pathogenic or likely pathogenic variant. Limitations include heterogeneity in genetic testing across cohorts, incomplete mutational characterization (eg, missed copy-number variant detection), and a predominance of participants of European ancestry, which may affect generalizability.

These findings suggest that current AAO-based recommendations, particularly restricting testing to AAO 50 years and younger, fail to identify a substantial proportion of patients with PD and pathogenic variants, including many carriers of *LRRK2* and *GBA1* variants who are the current focus of gene-specific trials. Broader genetic testing strategies that extend beyond early-onset and positive FH may better align clinical practice with emerging gene-specific therapies, and older age alone should not preclude testing where resources permit. Depending on resources and health care structures, such strategies could include raising the AAO threshold, offering panel testing to all patients with PD regardless of age, or combining AAO with clinical or biomarker features to prioritize testing in constrained settings. Our data-driven estimates of sensitivity and PPV across age groups can serve as a quantitative basis for revising current, expert opinion–based recommendations toward more age-inclusive genetic testing in PD.

## Notes

Supplement 1. eMethods 1. Cohorts and genetic testing eMethods 2. Statistical analyses eFigure. Flowchart of the exclusion of patients for the four individual datasets

Supplement 2. Members of the Global Parkinson’s Genetics Program (GP2), the ROPAD Study Group, and the PDGENEration Study

Supplement 3. Data sharing statement
