# Supplementary material for: Genetic Testing by Age at Onset in Parkinson Disease
Source: JAMA Neurol. 2026 May 11;83(7):711–3. doi: 10.1001/jamaneurol.2026.1112 (PMC13162140; doi:10.1001/jamaneurol.2026.1112)
Supplement: Supplement 1. — eMethods 1. Cohorts and genetic testing eMethods 2. Statistical analyses eFigure. Flowchart of the exclusion of patients for the four individual datasets [file jamaneurol-e261112-s001.pdf]

# Supplemental Online Content

Balck A, Vollstedt EJ, Westenberger A, et al; GP2; ROPAD study group; PDGENEration Study. Genetic testing by age at onset in parkinson disease. *JAMA Neurol*. Published online May 11, 2026. doi:10.1001/jamaneurol.2026.1112

**eMethods 1.** Cohorts and genetic testing
**eMethods 2.** Statistical analyses
**eFigure.** Flowchart of the exclusion of patients for the four individual datasets

This supplemental material has been provided by the authors to give readers additional information about their work.

© 2026 Balck A et al. *JAMA Neurology*.

## eMethods1: Cohorts and genetic testing

**ROPAD**

The Rostock Parkinson's Disease (ROPAD) study is a prospective, multicenter genetic screening study including over 12,000 individuals with a clinical diagnosis of PD from 16 countries. Genetic testing was performed in a diagnostic laboratory using a three-tiered protocol: targeted screening for common *LRRK2* variants and full *GBA1* sequencing, followed by next-generation sequencing of a predefined multigene panel, and, in selected cases with suspected monogenic PD, short-read whole-genome sequencing. Variants were interpreted according to ACMG/AMP criteria, and pathogenic, likely pathogenic, and risk variants were returned to participants with pre- and post-test counseling.

**PD GENEration**

PD GENEration is an ongoing, prospective, multicenter observational study led by the Parkinson's Foundation that offers free, clinical-grade genetic testing and counseling to individuals with PD in North America. Testing is performed in a CLIA- and CAP-certified laboratory using next-generation sequencing with exon-level coverage and copy-number variant analysis of seven PD-related genes (*GBA1*, *LRRK2*, *PRKN*, *PINK1*, *PARK7*, *SNCA*, *VPS35*). Variants are classified according to ACMG/AMP guidelines, and reportable results (pathogenic, likely pathogenic, or risk variants) are disclosed to participants.

**MDSGene, EPIPARK, and DeNoPa**

The MDSGene dataset is a curated, literature-based repository of individually annotated PD patients with pathogenic or probably pathogenic variants in *PRKN*, *PINK1*, *PARK7*, *LRRK2*, *SNCA*, *VPS35*, or *GBA1*. For this analysis, we complemented MDSGene with idiopathic PD (IPD) participants from the EPIPARK and DeNoPa cohort studies, who underwent panel-based testing for the same PD genes and were included as IPD controls only if no pathogenic or likely pathogenic variants were detected.

**GP2**

The Global Parkinson's Genetics Program (GP2) is a large, international genotyping and genome-sequencing initiative. For this project, we used genome-wide genotyping data from GP2 Release 9, excluding individuals already included in ROPAD, EPIPARK, or PD GENEration. Variants in *LRRK2*, *SNCA*, *VPS35*, *PRKN*, *PINK1*, *PARK7*, and *GBA1* were extracted and annotated, and only variants classified as pathogenic or likely pathogenic in ClinVar and/or under ACMG criteria were included; copy-number variants were not assessed.

**Phenotypes and predictors**

Across all datasets, genetic PD was defined as PD with two pathogenic or likely pathogenic variants in *PRKN*, *PINK1*, *PARK7*, or one pathogenic variant in *LRRK2*, *SNCA*, *VPS35*, or *GBA1*; all other PD patients were classified as idiopathic PD. Age at onset (AAO) was defined as the age at which the first cardinal motor symptom of PD occurred. Family history (FH) was coded as positive if at least one first-degree relative (PD GENEration) or any biologically related family member (other datasets) had PD. Patients with missing AAO, missing FH, or inconclusive genetic results (benign variants or variants of uncertain significance in the genes of interest) were excluded from the primary analyses. Individuals with pathogenic or likely pathogenic variants in more than one PD gene were also excluded to avoid ambiguity in assigning genetic PD status (**eFigure 1**).

## eMethods 2: Statistical analyses

We used receiver operating characteristic (ROC) analyses to evaluate AAO as a predictor of genetic PD vs IPD and estimated areas under the curve (AUCs) with 95% confidence intervals. Logistic regression models combining AAO and FH were used to obtain AUCs for the composite predictor; DeLong tests were applied to compare correlated AUCs for AAO alone vs AAO plus FH. Sensitivity, specificity, and positive predictive values (PPVs) were calculated for AAO thresholds (e.g., AAO ≤50) and for predefined AAO brackets; PPV estimation was restricted to ROPAD and PD GENEration, where variant prevalence in the tested populations was known. Gene-specific analyses contrasted findings in patients with recessive early-onset PD genes (*PRKN*, *PINK1*,

© 2026 Balck A et al. *JAMA Neurology*.

*PARK7*), *LRRK2*, and *GBA1* with IPD patients to assess gene-group–specific performance of AAO-based criteria.

**eFigure1: Flowchart of the exclusion of patients for the four individual datasets.** IPD = Idiopathic Parkinson's disease

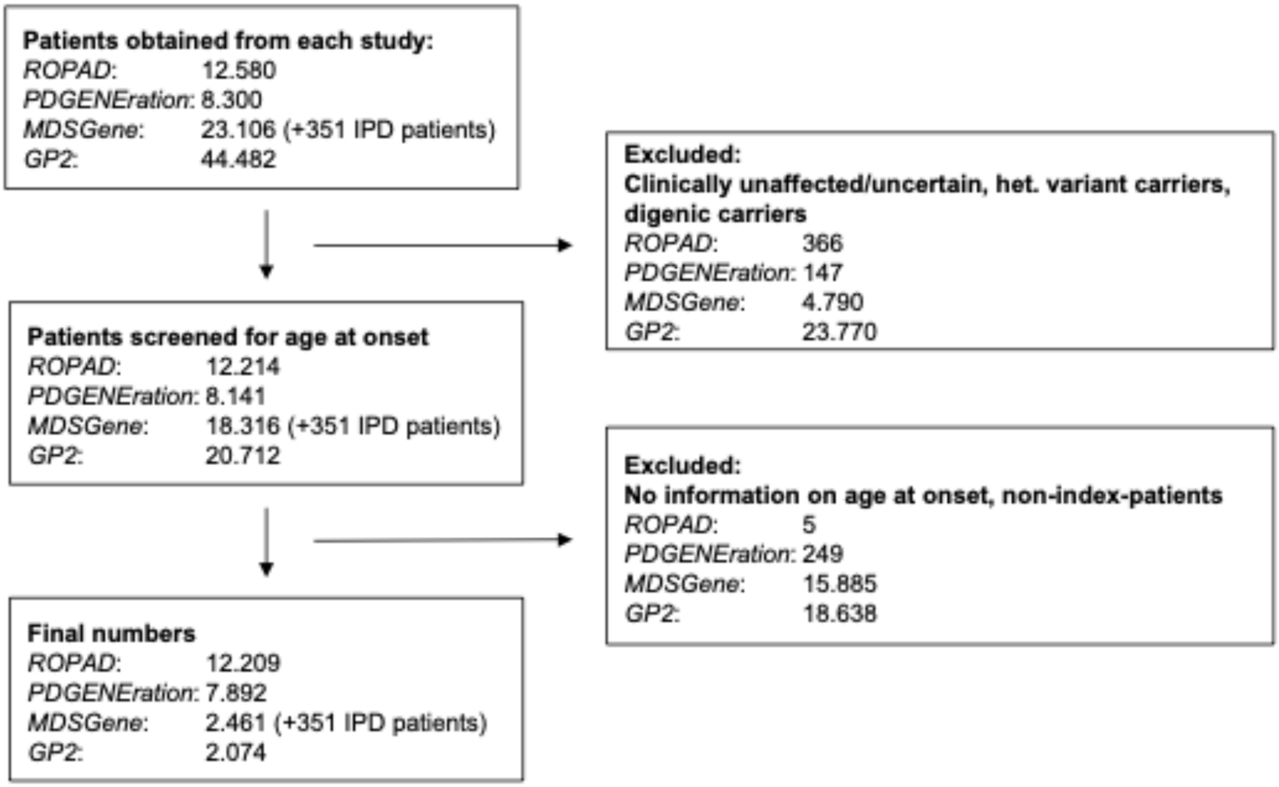


© 2026 Balck A et al. *JAMA Neurology*.
