# Supplementary material for: Genetic Testing by Age at Onset in Parkinson Disease
Source: JAMA Neurol. 2026 May 11;83(7):711–3. doi: 10.1001/jamaneurol.2026.1112 (PMC13162140; doi:10.1001/jamaneurol.2026.1112)
Supplement: Supplement 2. — Members of the Global Parkinson’s Genetics Program (GP2), the ROPAD Study Group, and the PDGENEration Study [file jamaneurol-e261112-s002.pdf]

*Group Name(s): Global Parkinson's Genetics Program (GP2), the ROPAD Study Group, and the PDGENEration Study								
***First Name and Middle Initial(s)**	***Last Name**	***Suffix (eg, Jr, III)**	Academic Degrees	Institution	Location (city, state/province, country)	Role or Contribution, eg, chair, principal investigator	Group (if more than 1 Group listed in the byline) and/or Subgroup (eg, Steering Committee)	
Whitley	Aamodt			University of Pennsylvania	Philadelphia		GP2	
Jan Olav	Aasly			Department of Neurology, St. Olavs Hospital, 7006 Trondheim, Trøndelag, Norway Department of Neuroscience, Norwegian University of Science and Technology, 7034 Trondheim, Norway			ROPAD	
Saltanat	Abdraimova			South Kazakhstan Medical Academy	Shymkent		GP2	
Fatimah	Abdulai			University of Abuja Teaching Hospita	Gwagwalada		GP2	
Aisuikum	Abdumalikova			I.K. Akhunbaev Kyrgyz State Medical Academy (KSMA), Osh City Clinical Hospital	Osh		GP2	
Mukhammadiusuf	Abdykadyrov			Osh State University	Osh		GP2	
Oladunni	Abiodun			General Hospital	Lagos		GP2	
Avigaelle	Abitbol			Hopital Universitaire Pitie Salpetriere, AP-HP	Paris		GP2	
Sani	Abubakar			Ahmadu Bello University	Kaduna State		GP2	
Charles	Achoru			Jos University Teaching Hospital	Jos		GP2	
Dario Sergio	Adamec			HOSPITAL NACIONAL PROFESOR	Buenos Aires		GP2	
Philip	Adebayo			The Aga Khan University	Dar es Salaam		GP2	
Charles H.	Adler			Mayo Clinic College of Medicine, Ma	Scottsdale		GP2	
Mitra	Afshari			Department of Neurological Sciences, Rush University Medical Center, 60612 Chicago,			ROPAD	
Osigwe	Agabi			College of Medicine, University of La	Lagos		GP2	
Vidhu	Agarwal			National Institute of Health	Bethesda		GP2	
Pinky	Agarwal		MD	Evergreen Health, Seattle, WA		Local Site Investigator	PDGENeration, ROPAD	
Yves	Agid			Paris Brain Institute	Paris		GP2	

*First Name and Middle Initial(s)	*Last Name	*Suffix (eg, Jr, III)	Academic Degrees	Institution	Location (city, state/province, country)	Role or Contribution, eg, chair, principal investigator	Group (if more than 1 Group listed in the byline) and/or Subgroup (eg, Steering Committee)	
Julian	Agin-Liebes		MD	Columbia University, New York, NY		National Recruiting Site Investigator; Local Site Investigator	PDGENeration	
Patricio Alejandro C	Aguilera			Universidad de Chile, Facultad de M	Santiago		GP2	
Uchechi	Agulanna			Lagos University Teaching Hospital	Lagos		GP2	
Azlina	Ahmad-Annuar			University of Malaya	Kuala Lumpur		GP2	
Seitzhan	Aidarov			National Center for Neurosurgery	Astana		GP2	
Sadenova	Aigul			Multidisciplinary City Hospital No. 1	Moscow		GP2	
Muhittin Cenk	Akbostancı			Private Practice	Ankara		GP2	
Gulnara	Akhmadeeva			Ufa Scientific Center	Ufa		GP2	
Vadim	Akhmetzhanov			Medline medical center	Astana		GP2	
Rufus	Akinyemi			Neuroscience and Ageing Research	Ibadan		GP2	
Albert	Akpalu			University of Ghana Medical School	Accra		GP2	
Wemimo	Alaofin			University of Ilorin	Ilorin		GP2	
Roger	Albin			Universit of Michigan	Ann Arbor		GP2	
Roy N.	Alcalay		MD, MS	Tel Aviv Sourasky Medical Center, Tel Aviv, Israel, Columbia University, New York, NY		Principal Investigator; Local Site Investigator; Steering Committee	PDGENeration, GP2	
Sarael	Alcauter		PhD	Universidad Autónoma de México, Santiago de Querétaro, QRO, Mexico		LATAM Site Investigator	PDGENeration, GP2	
Jason	Aldred		MD	Inland Northwest Research, Spokane, WA		Local Site Investigator	PDGENeration, ROPAD	
Fernando	Alonso-Frech			Department of Neurology, Movement Disorders Unit, Hospital Clínico San Carlos, 2804			ROPAD	
Nada Abdullah	Altassan			King Faisal Specialist Hospital and Research Center	Riyadh		GP2	
Griselda Judith	Alvarado			SIPROSA	Tucumán		GP2	
Ignacio	Alvarez			University Hospital Mutua Terrassa	Barcelona		GP2	
Dinara	Alzhanova			Astana Medical University	Astana		GP2	
Antonio Cristobal Lu	Ambrosiani			Instituto de Biomedicina de Sevilla	Sevilla		GP2	
Andrew Nader	AMeri			MUSC	Charleston		GP2	
Ferzana	Amod			University of KwaZulu-Natal	Durban		GP2	

Supplemental Online Content: Nonauthor Collaborators
*First name, last name, and suffix (if applicable) are required and will appear in PubMed.

*First Name and Middle Initial(s)	*Last Name	*Suffix (eg, Jr, III)	Academic Degrees	Institution	Location (city, state/province, country)	Role or Contribution, eg, chair, principal investigator	Group (if more than 1 Group listed in the byline) and/or Subgroup (eg, Steering Committee)	
Rim	Amouri			Mongi Ben Hmida National Institute of Neurolog	Tunis		GP2	
Tim J.	Anderson			University of Otago	Dunedin		GP2	
Roderick	Anderson			Tucson Neuroscience Research, 85710 Tucson, Arizona, USA			ROPAD	
Charlotte	Andrews			Queen Mary University of London	London		GP2	
Mathieu	Anheim			University Hospital of Strasbourg, Strasbourg, France	STRASBOURG		GP2	
Ifeyinwa	Ani-Osheku			Asokoro District Hospital	Abuja		GP2	
Grazia	Annesi			National Research Council	Cosenza		GP2	
Roosevelt	Anyanwu			College of Medicine, University of La	Lagos		GP2	
Rui	Araújo			Department of Neurology, Centro Hospitalar Universitário de São João, 4200-319 Porto, Porto District, Portugal Department of Clinical Neurosciences and Mental Health, Faculty of Medicine, University of Porto, 4200-319 Porto, Porto District, Portugal			ROPAD	
Gonzalo	Arboleda			Universidad Nacional de Colombia	Bogotá		GP2	
Alpher Perez	Arias			Clinica Union Medica del Norte	Santiago de los Caballeros		GP2	
David	Arkadir			Department of Neurology, Faculty of Medicine, Hadassah Medical Organization, Hebre			ROPAD	
Isabelle	Arnulf			Hopital Universitaire Pitie Salpetriere, AP-HP	Paris		GP2	
Lorena Hudson	Aros			INMOV (Instituto Nacional de Movimientos Anormales)	Buenos Aires		GP2	
Ana Paula Angulo	Arrieta			Hospital Angeles Puebla, Universidad Anáhuac Puebla	Puebla		GP2	
Shokhista	Artyshova			Clinical Hospital of the Presidential Administration of the Kyrgyz Republic	Bishkek		GP2	
Tatiana	Ascencio			Dr. Andres Bello university	San Salvador		GP2	
Sidra	Aslam			Banner Health	Phoenix		GP2	
Masharip	Atadzhanov			University of Zambia	Lusaka		GP2	

*First Name and Middle Initial(s)	*Last Name	*Suffix (eg, Jr, III)	Academic Degrees	Institution	Location (city, state/province, country)	Role or Contribution, eg, chair, principal investigator	Group (if more than 1 Group listed in the byline) and/or Subgroup (eg, Steering Committee)	
Nargiza	Atambekova			I.K. Akhunbaev Kyrgyz State Medical Academy (KSMA), Clinical Hospital of Emergency Medical Care	Bishkek		GP2	
Deborah	Attuah			YLD	London		GP2	
Elena	AVALE			CONICET	Buenos Aires		GP2	
Micol	Avenali			IRCCS Mondino Foundation, Neurogenetics Research Center, 27100 Pavia, Italy Department of Brain and Behavioral Sciences, University of Pavia, 27100 Pavia, Lombardy, Italy	Pavia		ROPAD, GP2	
Cesar Luis	Avila			CONICET-UNT	San Miguel de Tucumán		GP2	
Paula Saffie	Awad			Universidade Federal do Rio Grande do Sul	Porto Alegre		GP2	
Ignacio	Azcarate			Parkinson’s Foundation, New York, NY		Core Team	PDGENeration	
Shahrul	Azmin			Universiti Kebangsaan Malaysia Medical Centre	Kuala Lumpur		GP2	
Mehmet	Balal			Department of Neurology, School of Medicine, Çukurova University, 01330 Adana, Ada			ROPAD	
Alexander	Balck			University of Lübeck	Lübeck		GP2	
Shannon	Ballard			Data Tecnica	Bethesda		GP2	
Sabina	Baltabaeva			I.K. Akhunbaev Kyrgyz State Medical Academy (KSMA), Medcenter.kg" clinic	Bishkek		GP2	
Solomiia	Bandrivska			University College London	London		GP2	
Senaka	Bandusena			National Hospital of Sri Lanka	Colombo		GP2	
Gintare	Baranauskiene			Vilnius University	Vilnius		GP2	
Karla Salinas	Barboza			HOSPITAL GENERAL DE MEXICO	Mexico City		GP2	
Soraya	Bardien			Stellenbosch University	Stellenbosch		GP2	
Roger	Barker			University of Cambridge	Cambridge		GP2	
Matthew	Barrett		MD	Virginia Commonwealth University, Richmond, VA		Local Site Investigator	PDGENeration	

Supplemental Online Content: Nonauthor Collaborators
*First name, last name, and suffix (if applicable) are required and will appear in PubMed.

*First Name and Middle Initial(s)	*Last Name	*Suffix (eg, Jr, III)	Academic Degrees	Institution	Location (city, state/province, country)	Role or Contribution, eg, chair, principal investigator	Group (if more than 1 Group listed in the byline) and/or Subgroup (eg, Steering Committee)	
Cassandra	Barrett			The Michael J Fox Foundation	New York		GP2	
Deborah	Bartholdi			University Hospital Bern	Bern		GP2	
A. Nazlı	Başak			Koç University	Istanbul		GP2	
Peter	Bauer			CENTOGENE GmbH, 18055 Rostock, Mecklenburg-Vorpommern, Germany			ROPAD, GP2	
Thomas	Beach			Sun Health Research Institution	Sun City		GP2	
James C.	Beck		PhD	Parkinson's Foundation, New York, NY		Study Investigator; Steering Committee; Core Team	PDGENeration, GP2	
Christian	Beetz			CENTOGENE GmbH, 18055 Rostock, Mecklenburg-Vorpommern, Germany			ROPAD, GP2	
Nurayim	Beishembieva			I.K. Akhunbaev Kyrgyz State Medical Academy (KSMA)	Bishkek		GP2	
Samir	Bekadar			Eli Lilly and Company	Paris		GP2	
Erica	Bell			The Ohio State University Medical Center	Columbus		GP2	
Rafael Díaz	Belloso			Instituto de Biomedicina de Sevilla	Sevilla		GP2	
Eve	Benchetrit			La Timone Hospital	Marseille		GP2	
Sandra	Benizri			Movement Disorders Unit, Assuta Ramat Ha Hayal Hospital, 69710 Tel Aviv, Tel Aviv Di			ROPAD	
Wilma Van De	Berg			Vanderbilt University Medical Center	Amsterdam		GP2	
Daniela	Berg			University Medical Center Schleswig	Lübeck		GP2	
Maia	Beridze			Tbilisi State Medical University	Tbilisi		GP2	
Amelie	Bernardo			Hopital Universitaire Pitie Salpetriere, AP-HP	Paris		GP2	
Sagari	Bette			Parkinson's Disease and Movement Disorders Center of Boca Raton, 33486 Boca Ratoı			ROPAD	
Katrin	Beyer			Research Institute Germans Trias i Pujol	Barcelona		GP2	
Perminer	Bhatia			Neuro Pain Medical Center, 93720 Fresno, California, USA			ROPAD	
Kailash	Bhatia			University College London	London		GP2	
Basar	Bilgic			Istanbul University, Faculty of Medicine	Istanbul		GP2	
Andrew Jacobs	Bilson			Kwame Nkrumah University of Science and Technology	Kumasi		GP2	
Alexander	Blanski			Van Andel Research Institute	Grand Rapids		GP2	
Cornelis	Blauwendraat			Aligning Science Across Parkinson's (ASAP)	Bethesda		GP2	

Supplemental Online Content: Nonauthor Collaborators
*First name, last name, and suffix (if applicable) are required and will appear in PubMed.

*First Name and Middle Initial(s)	*Last Name	*Suffix (eg, Jr, III)	Academic Degrees	Institution	Location (city, state/province, country)	Role or Contribution, eg, chair, principal investigator	Group (if more than 1 Group listed in the byline) and/or Subgroup (eg, Steering Committee)	
Bas	Bleom			Radboud University	Nijmegen		GP2	
Karen	Blindauer		MD	Medical College of Wisconsin, Milwaukee, WI		Local Site Investigator	PDGENeration	
Bradley	Boeve			Mayo Clinic	Rochester		GP2	
Nicolaas I.	Bohnen			University of Michigan	Ann Arbor		GP2	
Michael	Bonello			Department of Neurology, The Walton Centre NHS Foundation Trust, L9 7LJ Liverpool,			ROPAD	
Dayany Leonel	Boone			Universidade Federal de São Paulo - UNIFESP/EPM	São Paulo		GP2	
Vanderci	Borges			Universidade Federal de São Paulo UNIFESP/EPM	São Paulo		GP2	
Per	Borghammer			Aarhus University	Aarhus		GP2	
Rupam	Borgohain			Nizam's Institute Of Medical Sciences	Hyderabad		GP2	
Nicola	Bothwick			Parkinson's Foundation, New York, NY		Core Team	PDGENeration	
Ahmed	Bouhouche			Specialties Hospital, CHU Ibn Sina	Rabat		GP2	
Iro	Boura			University of Crete	Heraklion		GP2	
Roberta	Bovenzi			University of Tor Vergata	Rome		GP2	
Jennifer	Brady			University College London	London		GP2	
Pedro	Braga-Neto			Division of Neurology, Department of Clinical Medicine, Federal University of Ceará, 60430-140 Fortaleza, Brazil<br>Center of Health Science, Universidade Estadual do Ceará, 60714-903 Fortaleza, Ceará, Brazil			ROPAD	
Chantale	Branson		MD	Morehouse College, Atlanta, GA	Atlanta	Local Site Investigator	PDGENeration, GP2	
Sarah	Brauneis			Inland Northwest Research, 99202 Spokane, Washington, USA			ROPAD	
David P.	Breen			Centre for Clinical Brain Sciences, University of Edinburg, 49 Little France Crescent, EH16 4SB Edinburgh, UK			ROPAD	

*First name, last name, and suffix (if applicable) are required and will appear in PubMed.

*First Name and Middle Initial(s)	*Last Name	*Suffix (eg, Jr, III)	Academic Degrees	Institution	Location (city, state/province, country)	Role or Contribution, eg, chair, principal investigator	Group (if more than 1 Group listed in the byline) and/or Subgroup (eg, Steering Committee)	
Noa	Bregman			Tel Aviv Medical Center	Tel Aviv-Yafo		GP2	
Susan	Bressman		MD	Mount Sinai Beth Israel Medical Center, New York, NY		Local Site Investigator	PDGENeration	
Alexis	Brice			Paris Brain Institute	Paris		GP2	
Hugo Morales	Briceño			Westmead Hospital	Sydney		GP2	
Andrea	Bril			Hospital Fernández	Buenos Aires		GP2	
Kathrin	Brockmann			University of Tübingen	Tübingen		GP2	
Kajsa	Brolin			Lund University	Lund		GP2	
Jeff	Bronstein		MD, PhD	University of California, Los Angeles (UCLA), Los Angeles, CA		National Recruiting Site Investigator; Local Site Investigator	PDGENeration	
Debi	Brooks			The Michael J Fox Foundation	New York		GP2	
Norbert	Brüggemann			University of Lübeck	Lübeck		GP2	
Michiko	Bruno		MD	Queen's Health System, Honolulu, HI	Honolulu	Local Site Investigator	PDGENeration, GP2	
Antonia Maria	Buchal			University Hospital Bonn	Bonn		GP2	
Jolanda	Buonocore			Magna Graecia University	Catanzaro		GP2	
David	Burn			Newcastle University	Newcastle upon Tyne		GP2	
María Leonor	Bustamante			Faculty of Medicine Universidad de Chile	Santiago		GP2	
Bernabé	Bustos			Northwestern University Feinberg School of Medicine	Chicago		GP2	
Lark	Caboy			Parkinson's Foundation, New York, NY		Core Team	PDGENeration	
Valentina	Caceres		MS, CGC	Indiana University, Indianapolis, IN		Genetics Counseling Core	PDGENeration	
Özgür Öztop	Çakmak			Koç University	Istanbul		GP2	
Manjula	Caldera			Teaching Hospital Anuradhapura	Anuradhapura		GP2	
Cristina Pérez	Calvo			Instituto de Biomedicina de Sevilla	Seville		GP2	
Sarah	Camargos			Universidade Federal de Minas Gerais	Belo Horizonte		GP2	
Francisca	Canals			Inmov	Santiago		GP2	
Dante Bernardo Orc	Canto			Hospital Angeles de Puebla	Puebla		GP2	

*First name, last name, and suffix (if applicable) are required and will appear in PubMed.

*First Name and Middle Initial(s)	*Last Name	*Suffix (eg, Jr, III)	Academic Degrees	Institution	Location (city, state/province, country)	Role or Contribution, eg, chair, principal investigator	Group (if more than 1 Group listed in the byline) and/or Subgroup (eg, Steering Committee)	
Fangyuan	Cao			QIMR Berghofer	Brisbane		GP2	
Federico	Capparelli			Centro de Educación Médica e Inves	Buenos Aires		GP2	
Jonathan	Carr			University of Stellenbosch	Stellenbosch		GP2	
Camille	Carroll			University of Plymouth	Plymouth		GP2	
Stephanie	Carvalho			Hopital Universitaire Pitie Salpetriere, AP-HP	Paris		GP2	
Bradford	Casey			The Michael J. Fox Foundation for Parkinson's Research	New York		GP2	
Juan Carlos Martíne	Castrillo			Hospital Ramón y Cajal	Madrid		GP2	
Francesco	Cavallieri			Neurology Unit, Neuromotor and Rehabilitation Department, Azienda Unità Sanitaria Locale-IRCCS di Reggio Emilia, 42122 Reggio Emilia, Emilia-Romagna, Italy			ROPAD	
Binnur	Çelik			University of Ankara	Ankara		GP2	
Amin	Cervantes-Arriaga			Instituto Nacional de Neurología y N	Mexico City		GP2	
Lana M.	Chahine			University of Pittsburgh	Pittsburgh		GP2	
Alizé	Chalançon			Hopital Universitaire Pitie Salpetriere, AP-HP	Paris		GP2	
Momodou	Cham			Richard Novati Catholic Hospital, Catholic Health Service Trust	Accra		GP2	
Piu	Chan			Capital Medical University	Beijing		GP2	
Phillip	Chan			The Hong Kong University of Science and Technology	Kowloon		GP2	
Germaine Hiu-Fai	Chan			Queen Elizabeth Hospital	Kowloon		GP2	
Pedro	Chaná		MD	Corporación Centro de Trastornos del Movimiento, Santiago, Chile		LATAM Site Investigator	PDGENeration	
Pedro	Chana-Cuevas			Universidad de Santiago de Chile	Santiago		GP2	
Shivika	Chandra			UTHealth-Houston	Houston		GP2	
Zach	Chaney			The Michael J Fox Foundation	New York		GP2	
Thashi	Chang			University of Colombo, Sri Lanka	Colombo		GP2	
Lisette	Charbonnier			Brain Research and Innovation Cent	Amsterdam		GP2	

Supplemental Online Content: Nonauthor Collaborators
*First name, last name, and suffix (if applicable) are required and will appear in PubMed.

*First Name and Middle Initial(s)	*Last Name	*Suffix (eg, Jr, III)	Academic Degrees	Institution	Location (city, state/province, country)	Role or Contribution, eg, chair, principal investigator	Group (if more than 1 Group listed in the byline) and/or Subgroup (eg, Steering Committee)	
Bruce A.	Chase			Endeavor Health (formerly NorthShore University Health System)	Skokie		GP2	
Honglei	Chen			Michigan State University	East Lansing		GP2	
Pak Leng	Cheong			Sydney Local Health District	Sydney		GP2	
Nelson Yuk-Fai	Cheung			Queen Elizabeth Hospital	Kowloon		GP2	
Elaine	Chew			Nanyang Technological University	Singapore		GP2	
Harneek	Chohan			Queen Mary University of London	London		GP2	
Kelvin	Chou		MD	University of Michigan, Ann Arbor, MI		Local Site Investigator	PDGENeration	
Sohini	Chowdhury			The Michael J. Fox Foundation for Parkinson's Research	New York		GP2	
Tesson	Christelle			Institut du Cerveau-Paris Brain Institute-ICM	Paris		GP2	
Brefel Courbon	Christine			CHU Toulouse	TOULOUSE		GP2	
Natalia	Chunga			Louisiana State University Health Sciences Center at Shreveport	Shreveport		GP2	
Joseph	Classen			Department of Neurology, Leipzig University Medical Center, 04103 Leipzig, Saxony, G			ROPAD	
Lisa	Cohen			Wake Research, 27612 Raleigh, North Carolina, USA			ROPAD	
Riaan Van	Coller			University of Pretoria	Pretoria		GP2	
Alicia	Colombo			University of Chile	Santiago		GP2	
Yaroslau	Compta			IDIBAPS / Hospital Clinic	Barcelona		GP2	
NS-PARK	Consortium			/	/		GP2	
Lola	Cook		MS, CGC	Indiana University, Indianapolis, IN		Genetics Counseling Core	PDGENeration	
Florence	Cormier-Dequaire			Hopital Universitaire Pitie Salpetriere, AP-HP	Paris		GP2	
Mario	Cornejo Olivas		MD	Instituto Nacional de Ciencias Neurológicas, Lima, Perú		LATAM Site Investigator	PDGENeration	
Mario	Cornejo-Olivas			Universidad Cientifica del Sur	Lima		GP2	
Ana Belen Miranda	Cortes			Fundación Diagnosis	Santiago		GP2	
Jean-Christophe	Corvol			Sorbonne Université	Paris		GP2	
Mariana Cavalcanti	Costa			Universidade Federal de São Paulo - UNIFESP/EPM	São Paulo		GP2	

*First name, last name, and suffix (if applicable) are required and will appear in PubMed.

*First Name and Middle Initial(s)	*Last Name	*Suffix (eg, Jr, III)	Academic Degrees	Institution	Location (city, state/province, country)	Role or Contribution, eg, chair, principal investigator	Group (if more than 1 Group listed in the byline) and/or Subgroup (eg, Steering Committee)	
Lucas Faria	Costa			Universidade Federal de Minas Gerais	Belo Horizonte		GP2	
Francisco Eduardo	Costa Cardoso			Movement Disorders Unit, Neurology Service, Department of Internal Medicine, Federal University of Minas Gerais, 31270-901 Belo Horizonte, Minas Gerais, Brazil			ROPAD	
Gist	Croft			The New York Stem Cell Foundation	New York		GP2	
David	Crosiers			Department of Neurology, Antwerp University Hospital, 2650 Edegem, Flemish, Belgium Translational Neurosciences, Faculty of Medicine and Health Sciences, University of Antwerp, 2610 Antwerp, Flemish, Belgium	Antwerp		ROPAD, GP2	
Carlos	Cruchaga			Washington University	St. Louis		GP2	
Rossy	Cruz Vicioso		MD	Unión Médica, Clínica Universitaria, Santiago de los Caballeros, Dominican Republic		LATAM Site Investigator	PDGENeration	
Karina	Cruz-Santillán			Instituto Nacional de Neurología y Neurocirugía	Mexico City		GP2	
Ilona	Csoti			Parkinson-Center, Gertrudisklinik Biskirchen, 35638 Leun, Hesse, Germany			ROPAD	
Esther	Cubo			Hospital Universitario Burgos	Burgos		GP2	
Paskal	Cullufi			Pediatric Department, University Hospital “Mother Teresa”, 1001 Tirana, Tirana Count			ROPAD	
Filipa	Curado			CENTOGENE GmbH, 18055 Rostock, Mecklenburg-Vorpommern, Germany			ROPAD	
Virginie	Czernecki			Hopital Universitaire Pitie Salpetriere, AP-HP	Paris		GP2	
Efthymios	Dadiotis			University of Thessaly	Volos		GP2	
Ioannis	Dagklis			Aristotle University of Thessaloniki	Thessaloniki		GP2	
Nabila	Dahodwala		MD	University of Pennsylvania, Philadelphia, PA	Philadelphia	Local Site Investigator	PDGENeration, GP2	
Kensuke	Daida			Juntendo University	Bunkyō		GP2	

Supplemental Online Content: Nonauthor Collaborators
*First name, last name, and suffix (if applicable) are required and will appear in PubMed.

***First Name and Middle Initial(s)**	***Last Name**	***Suffix (eg, Jr, III)**	Academic Degrees	Institution	Location (city, state/province, country)	Role or Contribution, eg, chair, principal investigator	Group (if more than 1 Group listed in the byline) and/or Subgroup (eg, Steering Committee)	
Aziza	Dalbaeva			National Hospital	Bishkek		GP2	
Solveig E J	Dalbro			Oslo University Hospital	Oslo		GP2	
John C.	Dalrymple-Alford			University of Canterbury	Christchurch		GP2	
Philippe	Damier			Nantes Université	Nantes		GP2	
Khashayar	Dashtipour			Department of Neurology, Division of Movement Disorders, Loma Linda University Sch			ROPAD	
Tom	Davis		MD	Vanderbilt University, Nashville, TN		Local Site Investigator	PDGENeration, GP2	
Ryan L	Davis			University of Sydney	Sydney		GP2	
Patricia	de Carvalho Aguiar			Department of Neurology and Neurosurgery, Hospital Israelita Albert Einstein, 05651-9			ROPAD	
Rebeca	De Leon			Parkinson’s Foundation, New York, NY		Core Team	PDGENeration	
Joy Antonelle	de Marcaida		MD	Hartford HealthCare, Hartford, CT		Local Site Investigator	PDGENeration	
Anna	De Rosa			Department of Neurosciences and Reproductive and Odontostomatological Sciences, F			ROPAD	
Marissa	Dean		MD	University of Alabama, Birmingham, Birmingham, AL	Birmingham	Local Site Investigator	PDGENeration, GP2	
Bertrand	Degos			Avicenne Hospital	Bobigny		GP2	
Laura Muñoz	Delgado			Instituto de Biomedicina de Sevilla	Sevilla		GP2	
Erick Gonzalez	Delgado			MedicalCenter Neurocenter S.A.	Santa Cruz de la Sierra		GP2	
Amanda	Deligtisch		MD	University of New Mexico, Albuquerque, NM		Local Site Investigator	PDGENeration	
Marcus Vinicius	Della Coletta			Department of Neurology, Universidade do Estado do Amazonas, 69050-010 Manaus A			ROPAD	
Meltem	Demirkiran			Department of Neurology, School of Medicine, Çukurova University, 01330 Adana, Ada			ROPAD	
Sumit	Dey			Queen Mary University of London	London		GP2	
Kamalesh	Dey			Queen Mary University of London	London		GP2	
Rohit	Dhall		MD	University of Arkansas, Fayetteville, AR		Local Site Investigator	PDGENeration	
Sergio García	Díaz			Instituto de Biomedicina de Sevilla	Sevilla		GP2	
Lola J.	Díaz-Feliz			Fernando Pessoa University, San Roque Hospital	Las Palmas de Gran Canaria		GP2	
Allison A.	Dilliott		PhD	Parkinson’s Foundation, New York, NY		Core Team	PDGENeration	
Paola	Dimartino			University of Pavia	Pavia		GP2	
Saiesha	Dindayal			University College London	London		GP2	

Supplemental Online Content: Nonauthor Collaborators
*First name, last name, and suffix (if applicable) are required and will appear in PubMed.

*First Name and Middle Initial(s)	*Last Name	*Suffix (eg, Jr, III)	Academic Degrees	Institution	Location (city, state/province, country)	Role or Contribution, eg, chair, principal investigator	Group (if more than 1 Group listed in the byline) and/or Subgroup (eg, Steering Committee)	
Megan	Dini		MA	Parkinson’s Foundation, New York, NY		Core Team	PDGENeration	
Elizabeth	Disbrow		PhD	Louisiana State University, Baton Rouge, LA	Shreveport	Local Site Investigator	PDGENeration, GP2	
Ruth	Djaldetti			Department of Neurology, Movement Disorders Clinic, Rabin Medical Center-Beilinson Hospital, 49100 Petach Tikva, Central District, Israel<br>Sackler Faculty of Medicine, Tel Aviv University, 6997801 Tel Aviv, Tel Aviv District, Israel			ROPAD	
Mouna Ben	Djebara			Razi Hospital	Tunis		GP2	
Pauline	Dodet			Hopital Universitaire Pitie Salpetriere, AP-HP	Paris		GP2	
Okan	Dogu			Department of Neurology, Mersin University, 33343 Mersin, Mersin Province, Turkey			ROPAD	
Carole	Dongmo-Kenfack			Hopital Universitaire Pitie Salpetriere, AP-HP	Paris		GP2	
Joseph	Donnelly			Te Whatu Ora - Health New Zealand Auckland, New Zealand	Auckland		GP2	
Maria Leila	Doquenia			Metropolitan Medical Center	Manila		GP2	
Charlotte	Dore			University College London	London		GP2	
Maria Gabriela	dos Santos Ghilardi			Laboratory of Neuroscience, Hospital Sírio-Libanês and Department of Neurology, University of São Paulo Medical School, 01308-050 São Paulo and 01246-903 São Paulo, São Paulo, Brazil			ROPAD	
Kirby	Doshier		PhD	Parkinson’s Foundation, New York, NY		Core Team	PDGENeration	
Erika	Driver-Dunckley			Mayo Clinic AZ	Scottsdale		GP2	
Felipe	Duarte-Zambrano			Cleveland Clinic	Cleveland		GP2	

Supplemental Online Content: Nonauthor Collaborators
*First name, last name, and suffix (if applicable) are required and will appear in PubMed.

*First Name and Middle Initial(s)	*Last Name	*Suffix (eg, Jr, III)	Academic Degrees	Institution	Location (city, state/province, country)	Role or Contribution, eg, chair, principal investigator	Group (if more than 1 Group listed in the byline) and/or Subgroup (eg, Steering Committee)	
Sonya	Dumanis			Aligning Science Across Parkinson's	Washington		GP2	
Reyna M.	Durón			Universidad Tecnológica Centroamericana	Tegucigalpa		GP2	
Alexandra	Durr			Paris Brain Institute	Paris		GP2	
Faiza	Durrani			Centre for Preventive Neurology	London		GP2	
Carsten	Eggers			Department of Neurology, University Hospital Marburg, 35037 Marburg, Hesse, Germany Department of Neurology, Knappschaftskrankenhaus Bottrop, 46242 Bottrop, North Rhine-Westphalia, Germany			ROPAD	
Sarah	El-Sadig			Faculty of medicine university of Khartoum	Khartoum		GP2	
Bulent	Elibol			Department of Neurology, Faculty of Medicine, Hacettepe University, 06100 Ankara, Ankara, Turkey			ROPAD	
Mohamed	Elkasaby			University Hospitals Cleveland Medical Center/Case Western Reserve University	Cleveland		GP2	
Aaron	Ellenbogen			Michigan Institute for Neurological Disorders, 48334 Farmington Hills, Michigan, USA Quest Research Institute, 48334 Farmington Hills, Michigan, USA			ROPAD	
Melina	Ellis			Concord Hospital	Concord		GP2	
Inas	Elsayed			Faculty of pharmacy University of Gezira	Wad Madani		GP2	
Murat	Emre			Istanbul university	Istanbul		GP2	
Cyril	Erameh			Irrua Specialist Teaching Hospital	Ilorin		GP2	
Joshua Ooi Chin	Ern			Queen Elizabeth Hospital	Kota Kinabalu		GP2	
Sibel	Ertan			Department of Neurology, Koç Unive	Istanbul		ROPAD, GP2	
Joaquim Aumatell	Escabies			IR SANT PAU	Barcelona		GP2	
Valentina	Escott-Price			Cardiff University	Cardiff		GP2	

Supplemental Online Content: Nonauthor Collaborators
*First name, last name, and suffix (if applicable) are required and will appear in PubMed.

*First Name and Middle Initial(s)	*Last Name	*Suffix (eg, Jr, III)	Academic Degrees	Institution	Location (city, state/province, country)	Role or Contribution, eg, chair, principal investigator	Group (if more than 1 Group listed in the byline) and/or Subgroup (eg, Steering Committee)	
Alberto J.	Espay			University of Cincinnati	Cincinnati		GP2	
Ingrid	Estmann		MD	Universidad Autónoma de Nuevo León, San Nicolás de los Garza, Mexico		LATAM Site Investigator	PDGENeration	
Ingrid	Estrada-Bellmann			UNIVERSITY HOSPITAL “DR JOSE E GONZALEZ”	Monterrey		GP2	
Mario	Ezquerra			FCRB-IDIBAPS	Barcelona		GP2	
Daniel	Ezuduemoih			Lagos University Teaching Hospital	Lagos		GP2	
Giorgio	Fabiani			Movement Disorders Unit, Hospital Angelina Caron, 83430-000 Curitiba, Paraná, Brazil			ROPAD	
Ory Magne	Fabienne			chu toulouse	toulouse		GP2	
Oriol De	Fabregues			Hospital Universitari Vall d'Hebron	Barcelona		GP2	
Stewart A	Factor			Emory University	Atlanta		GP2	
Faraz	Faghri			Data Tecnica	Bethesda		GP2	
Björn H.	Falkenburger			Department of Neurology, University Hospital and Faculty of Medicine Carl Gustav Car			ROPAD	
Zih-Hua	Fang			The German Center for Neurodegenerative Diseases	Göttingen		GP2	
Matt	Farrer			University of Florida College of Medi	Gainesville		GP2	
Simon	Farrow			Clinical Research Center of Nevada, 89119 Las Vegas, Nevada, USA			ROPAD	
Nabli Fatnassi	Fatma			National institute Mongi Ben Hmida of Neurology	Tunis		GP2	
Tsviya	Fay-Karmon			Sackler Faculty of Medicine, Tel Aviv University, 6997801 Tel Aviv, Tel Aviv District, Israel<br>Movement Disorders Institute and Department of Neurology, Chaim Sheba Medical Center, 52621 Ramat-Gan, Tel Aviv District, Israel			ROPAD	
Matilda Lily	Fenn			University College London	London		GP2	
Gerald J.	Ferencz			RWJBarnabas Health Medical Group, Shore Neurology, 08755 Toms River, New Jersey,			ROPAD	
Rosangela	Ferese			IRCCS Neuromed	Pozzilli		GP2	
Hubert	Fernandez		MD	Cleveland Clinic, Cleveland, OH		Local Site Investigator	PDGENeration	

*First Name and Middle Initial(s)	*Last Name	*Suffix (eg, Jr, III)	Academic Degrees	Institution	Location (city, state/province, country)	Role or Contribution, eg, chair, principal investigator	Group (if more than 1 Group listed in the byline) and/or Subgroup (eg, Steering Committee)	
Elias	Fernandez		MD	Universidad de Concepción, Concepción, Chile		LATAM Site Investigator	PDGENeration	
José Manuel	Fernández			INMOV	Buenos Aires		GP2	
Ruben	Fernandez-Santiago			Hospital Clínic de Barcelona	Barcelona		GP2	
Elias	Fernandez-Toledo			University of Concepción	Concepcion		GP2	
Arjuna	Fernando			National Hospital of Sri Lanka	Colombo		GP2	
Henrique Ballalai	Ferraz			Universidade Federal de São Paulo (UNIFESP/EPM)	São Paulo		GP2	
Jeanne	Feuerstein		MD	University of Colorado, Boulder, CO		Local Site Investigator	PDGENeration	
Katherine	Fiallos		CGC	Indiana University, Indianapolis, IN		Genetics Counseling Core	PDGENeration	
Andre	Fienemann			University of Lübeck	Lübeck		GP2	
Eugenia Morelos	Figaredo			ISSSTE Morelia	Morelia		GP2	
Spencer	Finch			Queen Mary University of London	London		GP2	
Megan	Finke			Parkinson's Foundation, New York, NY		Core Team	PDGENeration	
Brian	Fiske			The Michael J. Fox Foundation for Parkinson's Research	New York		GP2	
Nicholas	Fleming		MD	Atrium Health, Charlotte, NC		Local Site Investigator	PDGENeration	
Lina	Florentin			HYGEIA Hospital	Athens		GP2	
Edward	Fon			McGill University	Montreal		GP2	
Serena	Fong			Michael J. Fox Foundation	New York City		GP2	
Erich Talamoni	Fonoff			Laboratory of Neuroscience, Hospital Sírio-Libanês and Department of Neurology, University of São Paulo Medical School, 01308-050 São Paulo and 01246-903 São Paulo, São Paulo, Brazil			ROPAD	
Nahuel	Fonseca			Consejo Nacional de Investigaciones Científicas y Técnicas	Buenos Aires		GP2	

Supplemental Online Content: Nonauthor Collaborators
*First name, last name, and suffix (if applicable) are required and will appear in PubMed.

*First Name and Middle Initial(s)	*Last Name	*Suffix (eg, Jr, III)	Academic Degrees	Institution	Location (city, state/province, country)	Role or Contribution, eg, chair, principal investigator	Group (if more than 1 Group listed in the byline) and/or Subgroup (eg, Steering Committee)	
Jia Nee	Foo			Nanyang Technological University	Singapore		GP2	
Sylvie	Forlani			Hopital Universitaire Pitie Salpetriere, AP-HP	Paris		GP2	
Tatiana	Foroud		PhD	Indiana University, Indianapolis, IN	Indianapolis	Steering Committee; National Recruiting Site Investigator; Genetics Counseling Core	PDGENeration, GP2	
Erin	Foster			Washington University School of Medicine	St. Louis		GP2	
Yara Dadalti	Fragoso			Department of Neurology, Universidade Metropolitana de Santos, 11070-100 Santos S			ROPAD	
Joshua Luc Isherwo	Frost			UCL Queen Square Institute of Neurology	London		GP2	
Glenda Oliva	Fuentes			Fundación Lucas para la Salud	Tegucigalpa		GP2	
Riona	Fumi			University College London, Institute (	London		GP2	
Manabu	Funayama			Juntendo University	Tokyo		GP2	
Juan Cristobal Nuñe	Fuster			Hospital Clínico Unversidad de Chile - Clínica Alemana Santiago	Santiago		GP2	
Carolin	Gabbert			University of Lübeck	Lübeck		GP2	
Monica	Gagliardi			Magna Graecia University	Catanzaro		GP2	
Agata	Gajos			Medical University of Lodz	Lodz		GP2	
Ignacio Illán	Gala			IR SANT PAU	Barcelona		GP2	
Caterina	Galandra			IRCCS Mondino Foundation	Pavia		GP2	
Cécile	Galléa			ICM	Paris		GP2	
Benjamin Pizarro	Galleguillos			Centro de Imagenología, Hospital Clínico Universidad de Chile	Santiago		GP2	
Kamalini Ghosh	Galvelis			Parkinson's Foundation	Princeton		GP2	
Ziv	Gan-Or			McGill University	Montreal		GP2	
Harry	Gao		PhD	Fulgent Genetics, Temple City, CA		Genetics Testing Core	PDGENeration	
Daniel Macías	García			Instituto de Biomedicina de Sevilla	Sevilla		GP2	
Luis M.	García-Marín			Queensland Institute of Medical Res	Brisbane		GP2	
Anna	Gareeva			Ufa Federal Research Center	Ufa		GP2	
Pilar Gómez	Garre			Instituto de Biomedicina de Sevilla	Seville		GP2	

Supplemental Online Content: Nonauthor Collaborators
*First name, last name, and suffix (if applicable) are required and will appear in PubMed.

*First Name and Middle Initial(s)	*Last Name	*Suffix (eg, Jr, III)	Academic Degrees	Institution	Location (city, state/province, country)	Role or Contribution, eg, chair, principal investigator	Group (if more than 1 Group listed in the byline) and/or Subgroup (eg, Steering Committee)	
Alicia	Garrido			IDIBAPS-FCRB. Hospital Clinic Barcelona	Barcelona		GP2	
Thomas	Gasser			University of Tübingen	Tübingen		GP2	
Emilia M	Gatto			Sanatorio de la Trinidad Mitre-INEBA	Buenos Aires		GP2	
Rahul	Gaurav			Hopital Universitaire Pitie Salpetriere, AP-HP	Paris		GP2	
Gençer	Genç			Department of Neurology, Şişli Etfal	Istanbul		ROPAD, GP2	
Steve	Gentleman			Imperial College London	London		GP2	
Tatiana	Georgiades			University College London	London		GP2	
Kamalini	Ghosh Galvelis		MS	Parkinson's Foundation, New York, NY		Steering Committee; PDGENE Team Lead	PDGENeration	
Ro'ee	Gilron		PhD	Rune Labs, San Francisco, CA		Local Site Investigator	PDGENeration	
Irina	Gilyazova			Russian Academy of Sciences / Bas	Ufa		GP2	
Manon	Gomes			Hopital Universitaire Pitie Salpetriere, AP-HP	Paris		GP2	
Astrid Daniela Adar	Gómez			Instituto de Biomedicina de Sevilla	Sevilla		GP2	
Manuel Menendez	Gonzalez			Hospital Universitario Central de Asturias	Oviedo		GP2	
José Matías Arbelo	González			Hospital Universitario San Roque Las Palmas/ Universidad Fernando Pessoa Canarias (UFPC)	Las Palmas de Gran Canaria		GP2	
Arantza	Gorospe			Department of Neurology, de Navarra University Hospital, 31008 Pamplona, Navarre, S			ROPAD	
David	Grabli			Hopital Universitaire Pitie Salpetriere, AP-HP	Paris		GP2	
Francisco	Grandas			Movement Disorders Unit, University General Hospital Gregorio Marañón, 28007 Mad			ROPAD	
Spencer	Grant			National Institutes of Health	Bethesda		GP2	
Sergiu	Groppa			University of Mainz	Mainz		GP2	
Donald	Grosset			University of Glasgow	Glasgow		GP2	
Doreen	Gruber			Movement Disorders Clinic, 14547 Beelitz-Heilstätten, Brandenburg, Germany			ROPAD	
Mark	Gudesblatt			NYU Langone South Shore Neurologic Associates, 11751 Islip, New York USA			ROPAD	
Ana Castellano	Guerrero			Instituto de Biomedicina de Sevilla	Sevilla		GP2	

Supplemental Online Content: Nonauthor Collaborators
*First name, last name, and suffix (if applicable) are required and will appear in PubMed.

*First Name and Middle Initial(s)	*Last Name	*Suffix (eg, Jr, III)	Academic Degrees	Institution	Location (city, state/province, country)	Role or Contribution, eg, chair, principal investigator	Group (if more than 1 Group listed in the byline) and/or Subgroup (eg, Steering Committee)	
Kawmadi Wasunder	Gunawardena			Oxford University Hospitals NHS Foundation Trust	Oxford		GP2	
Aysegul	Gunduz			Istanbul University-Cerrahpasa, Cerrahpasa Faculty of Medicine	Istanbul		GP2	
Steven	Gunzler		MD	Case Western, Cleveland, OH		Local Site Investigator	PDGENeration	
Jifeng	Guo			Xiangya Hospital	Changsha		GP2	
Tanya	Gurevich			Movement Disorders Unit, Neurological Institute, Tel Aviv Sourasky Medical Center, Te			ROPAD	
Marie-Odile	Habert			Hopital Universitaire Pitie Salpetriere, AP-HP	Paris		GP2	
Georgios	Hadjigorgiou			University of Thessaly	Volos		GP2	
Johann	Hagenah			Department of Neurology, Westküstenklinikum Heide, 25746 Heide, Schleswig-Holstei			ROPAD	
Elodie	Hainque			Hopital Universitaire Pitie Salpetriere, AP-HP	Paris		GP2	
Deborah	Hall		MD	Rush University, Chicago, IL	Chicago	Local Site Investigator	PDGENeration, GP2	
Anne	Hall		MD	Parkinson's Foundation, New York, NY		Past Steering Committee	PDGENeration	
Hasmet A.	Hanagasi			Behavioral Neurology and Movement Disorders Unit, Department of Neurology, Istanbul Faculty of Medicine, Istanbul University, 34093 Istanbul, Istanbul, Turkey	Istanbul		ROPAD, GP2	
Ihtsham	Haq		MD	University of Miami, Miami, FL		Local Site Investigator	PDGENeration	
John	Hardy			University College London	London		GP2	
Bernhard	Haslinger			Technical University of Munich	Munich		GP2	
Sharon	Hassin		MD	Chaim Sheba Medical Center, Ramat Gan, Israel		Local Site Investigator	PDGENeration	

Supplemental Online Content: Nonauthor Collaborators
*First name, last name, and suffix (if applicable) are required and will appear in PubMed.

*First Name and Middle Initial(s)	*Last Name	*Suffix (eg, Jr, III)	Academic Degrees	Institution	Location (city, state/province, country)	Role or Contribution, eg, chair, principal investigator	Group (if more than 1 Group listed in the byline) and/or Subgroup (eg, Steering Committee)	
Sharon	Hassin-Baer			Sackler Faculty of Medicine, Tel Aviv University, 6997801 Tel Aviv, Tel Aviv District, Israel Movement Disorders Institute and Department of Neurology, Chaim Sheba Medical Center, 52621 Ramat-Gan, Tel Aviv District, Israel			ROPAD	
Nobutaka	Hattori			Juntendo University faculty of medicine	Tokyo		GP2	
Robert A.	Hauser			University of South Florida Parkinson's Disease and Movement Disorders Center of Exc			ROPAD	
Ann-Kathrin	Hauser			University of Tübingen	Tübingen		GP2	
Steven	He			Garvan Institute of Medical Research	Sydney		GP2	
Laura	Heathers			Indiana University, Indianapolis, IN		Genetics Counseling Core	PDGENeration	
Karl	Heilbron			Charité - Universitätsmedizin Berlin	Berlin		GP2	
Michael	Henderson			Van Andel Institute	Grand Rapids		GP2	
Conor	Hennessey			The Michael J Fox Foundation	New York		GP2	
Dena	Hernandez			National Institutes of Health	Bethesda		GP2	
Carlos	Hernandez			Universidad del Desarrollo	Santiago		GP2	
Alvaro	Hernandez			University of Costa Rica	San Jose		GP2	
Ana Jimena	Hernández-Medrano			Instituto Nacional de Neurología y N	Mexico City		GP2	
Jorge	Hernández-Vara			Neurology Department, Vall d'Hebron University Hospital, Universitat Autònoma de Ba			ROPAD	
Evelin Álvarez	Herrera			Universidad Tecnológica Centroamericana (UNITEC)	Tegucigalpa		GP2	
Birgit	Herting			Neurological Clinic, Diakonie-Klinikum Schwäbisch Hall, 74523 Schwäbisch Hall, Baden-			ROPAD	
Peter	Heutink			Global Parkinson's Genetics Program (GP2)	Pacifica		GP2	
Emily	Hill		MD	University of Cincinnati, Cincinnati, OH	Cincinnati	Local Site Investigator	PDGENeration, GP2	
Vanessa K.	Hinson			Department of Neurology, Medical U	Charleston		ROPAD, GP2	
Priscila	Hodges		MS, CGC	Indiana University, Indianapolis, IN		Genetics Counseling Core	PDGENeration	

Supplemental Online Content: Nonauthor Collaborators
*First name, last name, and suffix (if applicable) are required and will appear in PubMed.

*First Name and Middle Initial(s)	*Last Name	*Suffix (eg, Jr, III)	Academic Degrees	Institution	Location (city, state/province, country)	Role or Contribution, eg, chair, principal investigator	Group (if more than 1 Group listed in the byline) and/or Subgroup (eg, Steering Committee)	
Janet	Hoenicka			Institut de Recerca Sant Joan de Deu	Barcelona		GP2	
Elliot	Hogg			Department of Neurosurgery, Cedars-Sinai Medical Center, Movement Disorder Progra			ROPAD	
Günter	Höglinger			Department of Neurology, University Hospital, LMU Munich	Munich		GP2	
Anna	Hohler		MD	BMC Community Hospital, Boston, MA		Local Site Investigator	PDGENeration	
Matthias	Höllerhage			Hannover Medical School	Hannover		GP2	
Franziska	Hopfner			Department of Neurology, University Hospital, LMU Munich	Munich		GP2	
Mossafa	Hossain			Clinique OCEANIC	CASABLANCA		GP2	
Jean-Luc	Houeto			Limoges University Hospital	Limoges		GP2	
Henry	Houlden			University College London	London		GP2	
Marion	Houot			Hopital Universitaire Pitie Salpetriere, AP-HP	Paris		GP2	
Denise	Howting			Perron Institute for Neurological and Translational Science	Nedlands		GP2	
Sana	Hrir			University of Lübeck	Lübeck		GP2	
Michele T.	Hu			Nuffield Department of Clinical Neur	Oxford		ROPAD, GP2	
Eduardo	Hummelgen			Neurology Service, Hospital Angelina Caron, 83430-000 Curitiba, Paraná, Brazil			ROPAD	
Kelly	Hussey			University of South Florida Parkinson’s Disease and Movement Disorders Center of Exc			ROPAD	
Brook	Huxford			Queen Mary University of London	London		GP2	
Elena	Iakovenko			Research Center of Neurology	Moscow		GP2	
Laura	Ibanez			Washington University	St. Louis		GP2	
Khairul Azmi	Ibrahim			HOSPITAL SULTANAH NUR ZAHIRAH KUALA TERENGGANU	KUALA TERENGGANU		GP2	
Abdullahi	Ibrahim			Federal University of Health Science	Azare		GP2	
Farid	Ichou			Hopital Universitaire Pitie Salpetriere, AP-HP	Paris		GP2	
Jonas	Ihle			Hopital Triemli Zürich	Zürich		GP2	
Erica	Ikwenu			Lagos University Teaching Hospital	Lagos		GP2	
Maryenela	Illanes-Manrique			Instituto Nacional de Ciencias Neurologicas	Lima		GP2	
Anastasia	Illarionova			Deutsches Zentrum für Neurodegenerative Erkrankungen	Göttingen		GP2	

Supplemental Online Content: Nonauthor Collaborators
*First name, last name, and suffix (if applicable) are required and will appear in PubMed.

*First Name and Middle Initial(s)	*Last Name	*Suffix (eg, Jr, III)	Academic Degrees	Institution	Location (city, state/province, country)	Role or Contribution, eg, chair, principal investigator	Group (if more than 1 Group listed in the byline) and/or Subgroup (eg, Steering Committee)	
Zhyldyz	Imanalieva			I.K. Akhunbaev Kyrgyz State Medical Academy (KSMA), "Aqua Lab" LLC	Bishkek		GP2	
Frank	Imarhiagbe			University of Benin	Benin City		GP2	
Jon	Infante			Service of Neurology, University Hospital “Marqués de Valdecilla (IDIVAL)”, University of Cantabria, and “Centro de Investigación Biomédica en Red de Enfermedades Neurodegenerativas (CIBERNED)”, 39008 Santander, Cantabria, Spain			ROPAD	
Nancy	Ip			The Hong Kong University of Science and Technology	Kowloon		GP2	
Stuart	Isaacson		MD	Parkinson's Disease & Movement Disorders Center of Boca Raton, Boca Raton, FL		Local Site Investigator	PDGENeration, ROPAD	
Mariam	Isayan			Somnus Neurology Clinic	Yerevan		GP2	
Ismaila	Ishola			College of Medicine, University of La	Lagos		GP2	
Maksudjon	Isrofilov			Avicenna Tajik State Medical University	Dushanbe		GP2	
Adylbek	Iusupov			I.K. Akhunbaev Kyrgyz State Medical Academy (KSMA), Osh City Clinical Hospital	Osh		GP2	
Hirotaka	Iwaki			Data Tecnica	Washington		GP2	
Emmanuel	Iwuozo			Benue State University	Makurdi		GP2	
Kanan	Jafarov			Istanbul Klinik	Baku		GP2	
Joowon	Jang			Seoul National University	Seoul		GP2	
Joseph	Jankovic			Baylor College of Medicine	Houston		GP2	
Nancy Monroy	Jaramillo			Instituto Nacional de Neurología y N	Mexico City		GP2	
Simona	Jasaityte			University College London	London		GP2	
Serge	Jauma			Neurology Service, Hospital Universitari de Bellvitge, 08907 Barcelona, Catalonia, Spair			ROPAD	
Laetitia	Jeancolas			Telecom Sud Paris, Evry / ICM	Évry		GP2	
Marte	Jensen			University College London	London		GP2	

Supplemental Online Content: Nonauthor Collaborators
*First name, last name, and suffix (if applicable) are required and will appear in PubMed.

*First Name and Middle Initial(s)	*Last Name	*Suffix (eg, Jr, III)	Academic Degrees	Institution	Location (city, state/province, country)	Role or Contribution, eg, chair, principal investigator	Group (if more than 1 Group listed in the byline) and/or Subgroup (eg, Steering Committee)	
Beomseok	Jeon			Seoul National University Hospital	Seoul		GP2	
Lorena Clavijo	Jiménez			Instituto de Biomedicina de Sevilla	Sevilla		GP2	
Marlene	Jimenez-Del-Rio			Universidad de Antioquia	Medellín		GP2	
Alyssa	Johansson			Van Andel Institute	Grand Rapids		GP2	
Cabell	Jonas			Mid-Atlantic Permanente Medical Group	Bethesda		GP2	
Lietsel	Jones			Data Tecnica International	Bethesda		GP2	
Emily Navarro	Jones			University College London	London		GP2	
Ludmila	Jornea			Hopital Universitaire Pitie Salpetriere, AP-HP	Paris		GP2	
Heike Hesse	Joya			Universidad Tecnológica Centroamericana UNITEC	Tegucigalpa		GP2	
Suzanne	Judd			UAB	Birmingham		GP2	
Gulnar B.	Kabdrakhmanova			West Kazakhstan Marat Ospanov Medical University	Aktobe		GP2	
Nurbakyt	Kadyrov			Osh City Clinical Hospital	Osh		GP2	
Lim	Kai-Shi			University of Malaya	Kuala Lumpur		GP2	
Gulnaz	Kaishibayeva			Institute of Neurology and Neurorehabilitation	Almaty		GP2	
Rauan	Kaiyrzhanov			University College London	London		GP2	
Krishnakumar	Kandaswamy			Centogene GmBH	Berlin		GP2	
Prabesh	Kanel			University of Michigan	Ann Arbor		GP2	
Foivos S.	Kanellos			University of Ioannina	Ioannina		GP2	
Un	Kang			NYU Grossman School of Medicine	New York		GP2	
Chia Yuen	Kang			Hospital Queen Elizabeth	Kota Kinabalu		GP2	
Altynay	Karimova			Institute of Neurology and Neurorehabilitation	Almaty		GP2	
Marcelo	Kauffman		MD, PhD	Hospital Ramos Mejía, Buenos Aires, Argentina		LATAM Site Investigator	PDGENeration, GP2	
Mariam	Kekenadze			Tbilisi State Medical University	Tbilisi		GP2	
Ajantha	Keshavaraj			Teaching Hospital Jaffna	Jaffna		GP2	
Samson	Khachatryan			Somnus Neurology Clinic	Yerevan		GP2	
Talgat	Khaibullin			Semey Medical University	Semey		GP2	
Alima A.	Khamidulla			West Kazakhstan Marat Ospanov Medical University	Aktobe		GP2	

***First Name and Middle Initial(s)**	***Last Name**	***Suffix (eg, Jr, III)**	Academic Degrees	Institution	Location (city, state/province, country)	Role or Contribution, eg, chair, principal investigator	Group (if more than 1 Group listed in the byline) and/or Subgroup (eg, Steering Committee)	
Anna	Khamzina			I.K. Akhunbaev Kyrgyz State Medical Academy (KSMA), "Unimed" Clinic	Bishkek		GP2	
Tarannum	Khan		MD	Cleveland Clinic Weston, Weston, FL		Local Site Investigator	PDGENeration	
Irine	Khatiashvili			S. Khechinashvili University Hospital	Tbilisi		GP2	
Irine	Khatiashvilli			Ivane Javakhishvili Tbilisi State University	Tbilisi		GP2	
Fouad	Khoury			Université Saint George - Hôpital national de neurologie et de neurochirurgie	Beirut		GP2	
Yasaman	Kianirad		MD	University of Illinois-Chicago, Chicago, IL		Local Site Investigator	PDGENeration	
Karl	Kieburtz			Beth Israel Deaconess Medical Center	Boston		GP2	
Camilla	Kilbane			University Hospitals Cleveland Medical Center	Cleveland		GP2	
Annie	Killoran		MD	University of Iowa, Iowa City, IA		Local Site Investigator	PDGENeration	
Yun Joong	Kim			Yongin Severance Hospital	Seoul		GP2	
Ryul	Kim			SMG-SNU Boramae Meidcal Center	Seoul		GP2	
Jonggeol Jeff	Kim			Baylor College of Medicine	Bethesda		GP2	
HAN-Joon	Kim			Seoul National University Hospital	Seoul		GP2	
Thomas	Kimber			Central Adelaide Local Health Network	Adelaide		GP2	
Asha	Kishore			Aster Medcity	Kochi		GP2	
Victoria	Klee			The Ohio State University	Columbus		GP2	
Christine	Klein			University of Lübeck	Lübeck		GP2	
Teresa	Kleinz			University of Lübeck	Lübeck		GP2	
Martin	Klietz			Hannover Medical School	Hannover		GP2	
Sulev	Koks			Murdoch University	Perth		GP2	
Sushma	Kola		MD	Allegheny Health Network, Pittsburgh, PA		Local Site Investigator	PDGENeration	
Natalia	Koleva-Alazeh			Parkinson-Center, Gertrudisklinik Biskirchen, 35638 Leun, Hesse, Germany			ROPAD	

Supplemental Online Content: Nonauthor Collaborators
*First name, last name, and suffix (if applicable) are required and will appear in PubMed.

*First Name and Middle Initial(s)	*Last Name	*Suffix (eg, Jr, III)	Academic Degrees	Institution	Location (city, state/province, country)	Role or Contribution, eg, chair, principal investigator	Group (if more than 1 Group listed in the byline) and/or Subgroup (eg, Steering Committee)	
Morenikeji	Komolafe			Obafemi Awolowo University	Ile-Ife		GP2	
Inke	König			University of Lübeck	Lübeck		GP2	
Tsamis	Konstantinos			University of Ioannina	Ioannina		GP2	
Yoana	Kordovska			University College London	London		GP2	
Mathew	Koretsky			Data Tecnica	Bethesda		GP2	
Vikas	Kotagal			University of Michigan	Ann Arbor		GP2	
Divya	Kp			Sree Chitra Tirunal Institute for Medical Sciences and Technology	Thiruvananthapuram		GP2	
Paul	Krack			Inselspital Bern, University of Bern	Bern		GP2	
Rejko	Krüger			University of Luxembourg	Esch-sur-Alzette		GP2	
Konstantin	Kufer			German Centre for Neurodegenerative Diseases (DZNE) / University Hospital Bonn	Bonn		GP2	
Maggie	Kuhl			The Michael J. Fox Foundation for Parkinson's Research	New York		GP2	
Gregor	Kuhlenbäumer			Department of Neurology, University	Kiel		ROPAD, GP2	
Andrea	Kühn			Movement Disorder and Neuromodulation Unit, Department of Neurology, Charité, Ur			ROPAD	
Prashanth Lingappa	Kukkle			Manipal Hospital	Delhi		GP2	
Kishore	Kumar			Garvan Institute of Medical Research and Concord Repatriation General Hospital	Darlinghurst		GP2	
Pin-Jui	Kung			National Taiwan University	Taipei City		GP2	
Ashvin	Kuri			Queen Mary University of London	London		GP2	
Nicole	Kuznetsov			National Institute of Health	Bethesda		GP2	
Erika	Lam			University College London	London		GP2	
Christian	Lambert			Imperial College London	London		GP2	
Anthony	Lang			University of Toronto	Toronto		GP2	
Lara M.	Lange			Laboratory of Neurogenetics, National Institute on Aging/Institute of Neurogenetics, University of Luebeck	Bethesda		GP2	
Aymeric	Lanore			Paris Brain Institute	Paris		GP2	

Supplemental Online Content: Nonauthor Collaborators
*First name, last name, and suffix (if applicable) are required and will appear in PubMed.

*First Name and Middle Initial(s)	*Last Name	*Suffix (eg, Jr, III)	Academic Degrees	Institution	Location (city, state/province, country)	Role or Contribution, eg, chair, principal investigator	Group (if more than 1 Group listed in the byline) and/or Subgroup (eg, Steering Committee)	
Sarah	Lawrence		MS	Navitas Clinical Research, Rockville, MD		Site and Clinical Data Management Core; Steering Committee	PDGENeration	
Alejandra	Lázaro-Figueroa			National Autonomous University of M	Mexico City		GP2	
Mickael	Le			Hopital Universitaire Pitie Salpetriere, AP-HP	Paris		GP2	
Thiago Peixoto	Leal			Cleveland Clinic	Cleveland		GP2	
Jee-Young	Lee			SMG-SNU Boramae Medical Center, College of Medicine Seoul National University	Seoul		GP2	
Hamin	Lee			St George's, University of London	London		GP2	
Stéphane	Lehericy			Hopital Universitaire Pitie Salpetriere, AP-HP	Paris		GP2	
Juan Sebastián Sánc	León			Hospital de Clínicas de Porto Alegre	Porto Alegre		GP2	
Hampton	Leonard			Data Tecnica	Bethesda		GP2	
Elena Ojeda	Lepe			Instituto de Biomedicina de Sevilla	Sevilla		GP2	
Suzanne	Lesage			Paris Brain Institute (ICM)	Paris		GP2	
Grace Helena	Letro			Hospital da Pontificia Universiade Católica de Campinas (Hospital PUC-Campinas	Campinas		GP2	
James B	Leverenz			Cleveland Clinic	Cleveland		GP2	
Kristin S.	Levine			Data Tecnica	Washington		GP2	
Dana	Lewis			Aligning Science Across Parkinson's	Baltimore		GP2	
Susan	Li		MD, PhD	Navitas Clinical Research, Rockville, MD		Site and Clinical Data Management Core	PDGENeration	
Tsao-Wei	Liang		MD	Thomas Jefferson University, Philadelphia, PA		Local Site Investigator	PDGENeration	
Ingeborg Haugesag	Lie			Oslo University Hospital	Oslo		GP2	
Shen-Yang	Lim			University of Malaya	Kuala Lumpur		GP2	
Amanda	Lim			Queensland Institute of Medical Research (QIMR Berghofer)	Brisbane		GP2	
Chin-Hsien	Lin			National Taiwan University Hospital	Taipei City		GP2	

Supplemental Online Content: Nonauthor Collaborators
*First name, last name, and suffix (if applicable) are required and will appear in PubMed.

***First Name and Middle Initial(s)**	***Last Name**	***Suffix (eg, Jr, III)**	Academic Degrees	Institution	Location (city, state/province, country)	Role or Contribution, eg, chair, principal investigator	Group (if more than 1 Group listed in the byline) and/or Subgroup (eg, Steering Committee)	
Irene	Litvan		MD	University of California, San Diego, La Jolla, CA		Local Site Investigator	PDGENeration, ROPAD	
Zhenhua	Liu			Xiangya Hospital, Central South Univ	Changsha		GP2	
Laia Muñoz	Llahuna			IR Sant Pau	Barcelona		GP2	
Katja	Lohmann			University of Lübeck	Lübeck		GP2	
Sophia	Loiodice			Hopital Universitaire Pitie Salpetriere, AP-HP	Paris		GP2	
Bruno	Lopes Santos-Lobato			Department of Neurology, Hospital Ophir Loyola, 66050-380 Belem, Pará, Brazil			ROPAD	
Natalia	Lopez			Global Parkinson's Genetics Program (GP2)	Madrid		GP2	
Maria Fernanda	López			Hospital Nacional Alejandro Posadas. Argentina	El Palomar		GP2	
Tatiana	Lopez-Gonzalez			Universidad Nacional de Colombia	Bogotá		GP2	
Lydia	López-Manzanares			Department of Neurology, Movement Disorders Unit, La Princesa University Hospital, 2			ROPAD	
Ileana	Lorenzini			Banner Sun Health Research Institut	Sun City		GP2	
Naomi	Louie			The Michael J. Fox Foundation for Parkinson's Research	New York		GP2	
Seth	Love			University of Bristol	Bristol		GP2	
Yun	Lu		PhD	Navitas Clinical Research, Rockville, MD		Site and Clinical Data Management Core	PDGENeration	
Steven	Lubbe			Northwestern University	Chicago		GP2	
Defebvre	Luc			CHU Lille	Lille		GP2	
Boris	Lucero			Universidad Católica del Maule	Talca		GP2	
Torres	Luis			Instituto Nacional de Ciencias Neurologicas	Lima		GP2	
Nolia	Lumley			University College London	London		GP2	
Wei	Luo			Zhejiang University	Hangzhou		GP2	
Theresa	Lüth			University of Lübeck	Lübeck		GP2	
McKenzie	Luxmore			Department of Neurology, Duke University School of Medicine, 27710 Durham, North			ROPAD	
Elena	M.Dieguez			Facultad de Medicina universidad de de la tepublica montevideo uruguay	Montevideo		GP2	
Harutyun	Madoev			University of Lübeck	Lübeck		GP2	
Silvia Jesus	Maestre			Instituto de Biomedicina de Sevilla	Sevilla		GP2	

Supplemental Online Content: Nonauthor Collaborators
*First name, last name, and suffix (if applicable) are required and will appear in PubMed.

*First Name and Middle Initial(s)	*Last Name	*Suffix (eg, Jr, III)	Academic Degrees	Institution	Location (city, state/province, country)	Role or Contribution, eg, chair, principal investigator	Group (if more than 1 Group listed in the byline) and/or Subgroup (eg, Steering Committee)	
Mary B	Makarious			Data Tecnica	Bethesda		GP2	
Maria	Makrygianni			HYGEIA Hospital	Athens		GP2	
Irene Andonia	Malaty		MD	University of Florida, Gainesville, FL		Local Site Investigator	PDGENeration	
Sujeena	Manandhar			Evergreen Health Neuroscience Institute, 98034 Kirkland, Washington, USA			ROPAD	
Graziella	Mangone			Hopital Universitaire Pitie Salpetriere, AP-HP	Paris		GP2	
Ganieva	Manizha			Avicenna Tajik State Medical Univers	Dushanbe		GP2	
Jodi	Maple-Grødem			Stavanger University Hospital	Stavanger		GP2	
Veronique	Marcaud			Department of Neurology, Saint Joseph Hospital, 75014 Paris, Île-de-France, France			ROPAD	
Karen S.	Marder		MD, MPH	Columbia University, New York, NY		Past Steering Committee	PDGENeration	
Kenneth	Marek			Institute for Neurodegenerative Disorders	New Haven		GP2	
Zoltan	Mari		MD	Cleveland Clinic Las Vegas, Las Vegas, NV		Local Site Investigator	PDGENeration	
Louise-Laure	Mariani			Paris Brain Institute - Sorbonne University	Paris		GP2	
Makrygianni	Mariza			HYGEIA Hospital	Athens		GP2	
Katerina	Markopoulou			Department of Neurology, NorthShore University HealthSystem, 60201 Evanston, Illinois, USA Department of Neurology, Pritzker School of Medicine, University of Chicago, 60637 Chicago, Illinois, USA	Chicago		ROPAD, GP2	
Connie	Marras		MD, PhD	The Edmond J Safra Program in Parkinson’s Research, Toronto Western Hospital, University of Toronto, M5T 2S8 Toronto, Ontario, Canada	Toronto	Local Site Investigator	PDGENeration, ROPAD	
Luca	Marsili			University of Cincinnati	Cincinnati		GP2	

*First name, last name, and suffix (if applicable) are required and will appear in PubMed.

***First Name and Middle Initial(s)**	***Last Name**	***Suffix (eg, Jr, III)**	Academic Degrees	Institution	Location (city, state/province, country)	Role or Contribution, eg, chair, principal investigator	Group (if more than 1 Group listed in the byline) and/or Subgroup (eg, Steering Committee)	
Maria J	Marti			Hospital Clinic de Barcelona. Institut d'Investigacio Biomedica August Pi i Sunyer (IDIBAPS)	Barcelona		GP2	
Celia Painous	Marti			Hospital Clinic Barcelona	Barcelona		GP2	
Nicholas G.	Martin			QIMR Berghofer Medical Research Institute	Brisbane		GP2	
Nadia Alejandra Gai	Martinez			Centro Neurológico del Centro Médico ABC, Campus Santa Fe	Mexico City		GP2	
Miguel Inca	Martinez			Cleveland Clinic Foundation	Cleveland		GP2	
Susana Lissette Peñ	Martínez			UNAB	San Salvador		GP2	
Manuela San Eufras	Martínez			Instituto de Biomedicina de Sevilla	Sevilla		GP2	
Edith Janeth Gaspar	Martínez			Universidad Nacional Autónoma de I	Santiago de Querétaro		GP2	
Daniel	Martinez-Ramirez			Tecnologico de Monterrey	Monterrey		GP2	
Sonia Moreno	Masmela			Universidad de Antioquia	Medellín		GP2	
Marina	Mata			Hospital Universitario Infanta Sofia	Madrid		GP2	
Ignacio	Mata		PhD	Cleveland Clinic, Cleveland, OH	Cleveland	Steering Committee	PDGENeration, GP2	
Michele	Matarazzo			HM CINAC (Centro Integral de Neurociencias Abarca Campal), Fundación Hospitales de Madrid, Hospital Universitario HM Puerta del Sur, HM Hospitales, 28938 Madrid, Madrid, Spain			ROPAD	
Eliana Pineda	Mateus			Universidad del Rosario	Bogotá		GP2	
Ellen Hoven	Maurtveten			Oslo University Hospital	Oslo		GP2	
Ahmad Shahir Bin	Mawardi			Hospita Kuala Lumpur	Kuala Lumpur		GP2	
Patrick	May			University of Luxembourg	Esch-sur-Alzette		GP2	
Kathleen	McKee		MD	Intermountain Healthcare, Salt Lake City, UT		Local Site Investigator	PDGENeration	
Mark	McKenzie			ClinSearch, 37421 Chattanooga, Tennessee, USA			ROPAD	
Catriona	Mclean			Florey neuroscience	Melbourne		GP2	
Yasser	Mecheri			Centre Hospitalo-Universitaire Dr Benbadis Constantine	Constantine		GP2	
Alex	Medina			Hospital San Felipe	Tegucigalpa		GP2	
Marika	Megrelishvili			Ilia State University	Tbilisi		GP2	

*First name, last name, and suffix (if applicable) are required and will appear in PubMed.

*First Name and Middle Initial(s)	*Last Name	*Suffix (eg, Jr, III)	Academic Degrees	Institution	Location (city, state/province, country)	Role or Contribution, eg, chair, principal investigator	Group (if more than 1 Group listed in the byline) and/or Subgroup (eg, Steering Committee)	
Shyamal	Mehta			Mayo Clinic, Arizona	Scottsdale		GP2	
Niccolo	Mencacci		MD, PhD	Northwestern University, Chicago, IL	Evanston	Steering Committee; National Recruiting Site Investigator; Local Site Investigator	PDGENeration, GP2	
Karen Lizzette Velas	Mendez			Universidad Nacional de Colombia	Bogotá		GP2	
Yan	Meng		PhD	Fulgent Genetics, Temple City, CA		Genetics Testing Core	PDGENeration	
Shreya	Menon			Gladstone Institutes	San Francisco		GP2	
Poornima	Menon			Hopital Universitaire Pitie Salpetriere, AP-HP	Paris		GP2	
Marcelo	Merello			Movement Disorders Service FLENI, CONICET, C1428 Buenos Aires, Ciudad Autónoma (			ROPAD, GP2	
Sophie	Meyer			Queen Mary University of London	London		GP2	
Chokri	Mhiri			Habib Bourguiba University Hospital	Sfax		GP2	
Vesna Van	Midden			Ljubljana University Medical Centre	Ljubljana		GP2	
Amanda	Miller		MS, CGC	Indiana University, Indianapolis, IN		Genetics Counseling Core	PDGENeration	
Kelly	Mills		MD	Johns Hopkins University, Baltimore, MD		Local Site Investigator	PDGENeration	
Andona	Milovanović			Neurology Clinic, University Clinical Center of Serbia	Belgrade		GP2	
Pablo	Mir			Instituto de Biomedicina de Sevilla	Seville		GP2	
Marcelo	Miranda			Departament of Neurology, Clinica MEDS, Santiago , Chile . Fundación Diagnosis , Santiago , Chile	Santiago		GP2	
Nataša Dragašević	Mišković			Neurology Clinic, University Clinical Center of Serbia	Belgrade		GP2	
Christina	Missler			Van Andel Institute	Grand Rapids		GP2	
Janis	Miyasaki		MD	University of Alberta, Edmonton, AB		Local Site Investigator	PDGENeration	
Fanny	Mochel			Hopital Universitaire Pitie Salpetriere, AP-HP	Paris		GP2	

Supplemental Online Content: Nonauthor Collaborators
*First name, last name, and suffix (if applicable) are required and will appear in PubMed.

*First Name and Middle Initial(s)	*Last Name	*Suffix (eg, Jr, III)	Academic Degrees	Institution	Location (city, state/province, country)	Role or Contribution, eg, chair, principal investigator	Group (if more than 1 Group listed in the byline) and/or Subgroup (eg, Steering Committee)	
Wael	Mohamed			International Islamic University	Kuala Lumpur		GP2	
Muhammed Saamir	Mohideen			National Hospital Galle	Galle		GP2	
Kin Ying	Mok			Univeristy College London	London		GP2	
Brit	Mollenhauer			Paracelsus-Elena-Klinik, 34128 Kassel, Hesse, Germany Department of Neurology, University Medical Centre Göttingen, 37075 Göttingen, Lower Saxony, Germany	Göttingen		ROPAD, GP2	
Oury	Monchi			Institut universitaire de gériatrie de Montréal	Montreal		GP2	
Roger Rodriguez	Monge			Universidad de Costa Rica	San José		GP2	
Javier Pagonabarrag	Mora			IR SANT PAU	Barcelona		GP2	
John C.	Morgan			Movement & Memory Disorder Programs, Department of Neurology, Augusta Universi			ROPAD	
Huw R	Morris			University College London	London		GP2	
Christopher M	Morris			Newcastle University	Newcastle upon Tyne		GP2	
Mariam	Mshvenieradze			Ivane Javakhishvili Tbilisi State University	Tbilisi		GP2	
Bashayer Al	Mubarak			King Faisal Specialist Hospital and Research Center	Riyadh		GP2	
Aliia	Mukhanova			International Higher School of Medicine	Bishkek		GP2	
Maria Valentina	Muller			Hospital General San Martin	La Plata		GP2	
Stephen	Mullin			Institute of Translational and Stratified Medicine, University of Plymouth School of Me			ROPAD	
Esteban	Muñoz			Hospital Clínic de Barcelona	Barcelona		GP2	
Nor Azian Abdul	Murad			UKM Medical Molecular Biology Institute	Kuala Lumpur		GP2	
Eduardo Jose Ponce	Murillo			Universidad Tecnológica Centroamericana	Tegucigalpa		GP2	
Kaileigh	Murphy			The Michael J. Fox Foundation for Parkinson's Research	New York		GP2	
Thomas	Musacchio			Department of Neurology, University Hospital of Würzburg, 97080 Würzburg, Bavaria,			ROPAD	
Daniel Jeremy	Myall			New Zealand Brain Research Institution	Christchurch		GP2	
Bennet	Myers			DENT Neurologic Institute, 14226 Buffalo, New York, USA			ROPAD	

*First Name and Middle Initial(s)	*Last Name	*Suffix (eg, Jr, III)	Academic Degrees	Institution	Location (city, state/province, country)	Role or Contribution, eg, chair, principal investigator	Group (if more than 1 Group listed in the byline) and/or Subgroup (eg, Steering Committee)	
Zhanybek	Myrzayev			International University of Postgradu	Almaty		GP2	
Mike A.	Nalls			Data Tecnica	Bethesda		GP2	
Martha	Nance		MD	Park Nicollet Struthers Parkinson's Center, Minneapolis, MN		Steering Committee; Local Site Investigator	PDGENeration	
Anna	Negrotti			Department of General and Specialized Medicine, Neurology Unit, University Hospital			ROPAD	
Alia	Neibaur			Indiana University, Indianapolis, IN		Genetics Counseling Core	PDGENeration	
Pedro Braga	Neto			Federal University of Ceará	Fortaleza		GP2	
NguyễN Thái Thuỳ	Ngân			University Medical Center, Ho Chi Minh City	Ho Chi Minh		GP2	
Vinh Thanh	Nguyen			University Medical Center Ho Chi Minh City	Ho Chi Minh		GP2	
Toan	Nguyen			Hue University	Huế		GP2	
Duan	Nguyen			Hue University	Huế		GP2	
Melissa	Nicewaner			Parkinson’s Foundation, New York, NY		Core Team	PDGENeration	
Anette	Nieves			Renstar Medical Research, 34471 Ocala, Florida, USA			ROPAD	
David Fernando Agu	Niño			University of Antioquia	Medellín		GP2	
Melissa	Nirenberg			Icahn School of Medicine at Mount S	New York		GP2	
Zeev	Nitsan			Department of Neurology, Barzilai Medical Center, 78278 Ashkelon, Southern District, Israel Faculty of Health Sciences, Ben Gurion University of the Negev, 84105 Beer-Sheva, Southern District, Israel			ROPAD	
Alero	Nnama			University of Port Harcourt Teaching	Port Harcourt		GP2	
Mohamed Ibrahim	Norlinah			Universiti Kebangsaan Malaysia	Selangor		GP2	
Scott A.	Norris			Washington University	St. Louis		GP2	
Mohamed	Nour			Razi Hospital	Rawalpindi		GP2	
Alastair	Noyce			Queen Mary University of London	London		GP2	
Juan Cristobal	Nuñez			Universidad de Chile - Clínica Alemana Santiago	Santiago		GP2	

*First Name and Middle Initial(s)	*Last Name	*Suffix (eg, Jr, III)	Academic Degrees	Institution	Location (city, state/province, country)	Role or Contribution, eg, chair, principal investigator	Group (if more than 1 Group listed in the byline) and/or Subgroup (eg, Steering Committee)	
Bermet	Nurbekova			I.K. Akhunbaev Kyrgyz State Medical Academy (KSMA)	Bishkek		GP2	
Karen	Nuytemans			University of Miami Miller School of Medicine	Miami		GP2	
Paul	Nwani			Nnamdi Azikiwe University Teaching	Nnewi		GP2	
Francisca	Nwaokorie			University of Lagos	Lagos		GP2	
Ernest	Nwazor			Rivers State University Teaching Ho:	Port Harcourt		GP2	
Eunice	Nyambane			Aga Khan University Nairobi	Nairobi		GP2	
Alyssa	O'Grady			The Michael J. Fox Foundation for Parkinson's Research	New York		GP2	
John	O'Sullivan			University of Queensland	Brisbane		GP2	
Vida	Obese			Kwame Nkrumah University of Science and Technology	Kumasi		GP2	
Yahaya	Obiabo			Federal University of Health Science	Otukpo		GP2	
Nkechi	Obianozie			University of Abuja Teaching Hospita	Gwagwalada		GP2	
Victor Flores	Ocampo			QIMR Berghofer Medical Research Institute	Brisbane		GP2	
Olanike	Odeniyi			General Hospital	Lagos		GP2	
Francis	Odiase			University of Benin	Benin City		GP2	
Ewere Marie	Ogbimi			Delta State University	Abraka		GP2	
Natalia Soledad	Ogonowski			QIMR Berghofer	Brisbane		GP2	
Adebimpe	Ogunmodede			Federal Medical Center	Owo		GP2	
Rajeev	Ojha			Tribhuvan University	Kirtipur		GP2	
Francis	Ojini			University of Lagos	Lagos		GP2	
Oluwadamilola	Ojo			College of Medicine of the University of Lagos	Lagos		GP2	
Eleni	Okeanis Vaou		MD	University of Texas San Antonio (UTSA), San Antonio, TX		National Recruiting Site Investigator; Local Site Investigator	PDGENeration	
Kigocha	Okeng'o			Muhimbili National Hospital-Mloganzila	Dar es Salaam		GP2	
Chiamaka	Okereke			University of Nigeria Teaching Hospi	Ituku-Ozalla		GP2	
Njideka	Okubadejo			University of Lagos	Lagos		GP2	
Olaitan	Okunoye			University College London	London		GP2	

Supplemental Online Content: Nonauthor Collaborators
*First name, last name, and suffix (if applicable) are required and will appear in PubMed.

*First Name and Middle Initial(s)	*Last Name	*Suffix (eg, Jr, III)	Academic Degrees	Institution	Location (city, state/province, country)	Role or Contribution, eg, chair, principal investigator	Group (if more than 1 Group listed in the byline) and/or Subgroup (eg, Steering Committee)	
Rashidat	Olanigan			Lagos State University Teaching Hos	Ikeja		GP2	
Maira Rozenfeld	Olchik			Hospital de Clínicas de Porto Alegre (HCPA)	Porto Alegre		GP2	
Adedunni	Olusanya			College of Medicine, University of La	Lagos		GP2	
Gerald	Onwuegbuzie			University of Abuja	Abuja		GP2	
Jorge	Orozco			Fundación Valle del Lili	Santiago De Cali		GP2	
Jorge Luis	Orozco Vélez		MD	Fundación Valle del Lili, Cali, Colombia		LATAM Site Investigator	PDGENeration	
Godwin	Osaigbovo			Jos University Teaching Hospital	Jos		GP2	
Sarah	Osborne			Parkinson’s Foundation, New York, NY		Core Team	PDGENeration	
Nosakhare	Osemwegie			University of Port Harcourt	Port Harcourt		GP2	
Olajumoke	Oshinaike			Lagos State University College of Me	Ikeja		GP2	
Nader	Oskooilar			Pharmacology Research Institute, 92660 Newport Beach, California, USA			ROPAD	
Zhumagul	Osmonova			Kyrgyz State Medical Institute of postgraduate training and continuous education named after S.B. Daniyarov	Bishkek		GP2	
Beatriz Munoz	Ospina			Fundacion Valle del Lili	Santiago De Cali		GP2	
Jill	Ostrem		MD	University of California, San Francisco, San Francisco, CA		Local Site Investigator	PDGENeration	
Alejandro Salguero	Oviedo			Instituto de Biomedicina de Sevilla	Sevilla		GP2	
Raymond	Owolabi			Federal Medical Center	Owo		GP2	
Lukman	Owolabi			Bayero University Kano	Kano		GP2	
Shyngle	Oyakhire			National Hospital	Abuja		GP2	
Simon Izuchukwu	Ozomma			University of Calabar Teaching Hosp	Calabar		GP2	
Özgür	Öztop Çakmak			Department of Neurology, Koç University, 34450 Istanbul, Istanbul, Turkey			ROPAD	
Eduardo De	Pablo-Fernández			Queen Mary University of London	London		GP2	
Mahesh	Padmanaban			University of Chicago	Chicago		GP2	
Shalini	Padmanabhan			Michael J. Fox Foundation	New York City		GP2	
Rajesh	Pahwa		MD	University of Kansas, Lawrence, KS		Local Site Investigator	PDGENeration	
Maxine	Paige-Pritchett			Università di Pavia	Pavia		GP2	
Azalea Tenerife	Pajo			University of Malaya	Kuala Lumpur		GP2	

Supplemental Online Content: Nonauthor Collaborators
*First name, last name, and suffix (if applicable) are required and will appear in PubMed.

*First Name and Middle Initial(s)	*Last Name	*Suffix (eg, Jr, III)	Academic Degrees	Institution	Location (city, state/province, country)	Role or Contribution, eg, chair, principal investigator	Group (if more than 1 Group listed in the byline) and/or Subgroup (eg, Steering Committee)	
Pramod	Pal			National Institute of Mental Health & Neurosciences	Bengaluru		GP2	
Gian	Pal		MD	Department of Neurology, Rutgers Robert Wood Johnson Medical School, 08901 New Brunswick, New Jersey, USA		Local Site Investigator	PDGENeration, ROPAD	
Eduardo Pérez	Palma			Universidad del Desarrollo	Santiago		GP2	
Vala	Palmadottir			deCODE genetics/Amgen Inc., Reykjavik, Iceland Faculty of Medicine, University of Iceland, Reykjavik, Iceland	Reykjavik		GP2	
Ilaria	Palmieri			IRCCS Mondino Foundation	Pavia		GP2	
Floria Carla	Pancetti		MD, PhD	Universidad Católica del Norte, Coquimbo, Chile	Coquimbo	LATAM Site Investigator	PDGENeration, GP2	
Caroline B.	Pantazis			Coalition for Aligning Science	Bethesda		GP2	
Ariane	Park		MD	Ohio State University Medical Center, Columbus, OH	Columbus	National Recruiting Site Investigator; Local Site Investigator	PDGENeration, GP2	
Laura	Parkkinen			University of Oxford	Oxford		GP2	
Lucilla	Parnetti			University of Perugia	Perugia		GP2	
Pau	Pastor			University Hospital Germans Trias i Pujol	Barcelona		GP2	
Krutika	Patel			New York Stem Cell Foundation	Denver		GP2	
Gamini Karapitiya	Pathirana			National Hospital of Sri Lanka	Colombo		GP2	
Jefri J.	Paul			CENTOGENE GmbH, 18055 Rostock, Mecklenburg-Vorpommern, Germany			ROPAD	
Nicola	Pavese			Clinical Ageing Research Unit, Newcastle University, NE4 5PL, Newcastle Upon Tyne, Ty			ROPAD	
Haydeh	Payami			The University of Alabama at Birming	Birmingham		GP2	
Valentina Quintana	Pena			Icesi University	Cali		GP2	
Susana Lissette	Peña Martinez		MD	Universidad Dr. Andrés Bello, San Salvador, El Salvador		LATAM Site Investigator	PDGENeration	
Oscar	Peña-Rodas			Universidad Dr Andrés Bello	San Salvador		GP2	
Antonio	Percesepe			Department of Medicine and Surgery, University of Parma, 43126 Parma, Emilia-Roma			ROPAD	

Supplemental Online Content: Nonauthor Collaborators
*First name, last name, and suffix (if applicable) are required and will appear in PubMed.

*First Name and Middle Initial(s)	*Last Name	*Suffix (eg, Jr, III)	Academic Degrees	Institution	Location (city, state/province, country)	Role or Contribution, eg, chair, principal investigator	Group (if more than 1 Group listed in the byline) and/or Subgroup (eg, Steering Committee)	
Laura	Pérez-Carbonell			Queen Mary University London / Guy's and St Thomas' NHS Foundation Trust	London		GP2	
Maria Teresa	Periñan			Instituto de Biomedicina de Sevilla	Seville		GP2	
Maria Teresa Muño	Personal			Universidad de Chile	Santiago		GP2	
Dijana	Petrovska			Telecom Sud Paris, Evry	Évry		GP2	
Ryan	Pfingst			The Michael J Fox Foundation	New York		GP2	
Tommaso	Piccoli			Unit of Neurology, Department of Biomedicine, Neurosciences and advanced Diagnost			ROPAD	
Lasse	Pihlstrøm			Oslo University Hospital	Oslo		GP2	
Nikita	Pillay			University of the Western Cape	Bellville		GP2	
Chen	Pin-Shiuan			National Taiwan University Hospital	Taipei		GP2	
David Antonio	Pineda-Salazar			GRUPO DE NEUROCIENCIAS DE ANTIOQUIA (GNA)	Medellín		GP2	
María Eugenia Cont	Pinto			Hospital San Juan de Dios	La serena		GP2	
JULIA ESTHER RIOS	PINTO			UNIVERSIDAD PERUANA LOS ANDES	Huancayo		GP2	
Carolina	Pinto de Souza			Department of Neurology, São Francisco Hospital, University of São Paulo, 01236-030 S			ROPAD	
Toni L.	Pitcher			University of Otago	Dunedin		GP2	
Tymothée	Poitou			ICM	Paris		GP2	
John	Poma			Parkinson's Foundation, New York, NY		Steering Committee	PDGENeration	
Chatkaew	Pongmala			University of Michigan	Ann Arbor		GP2	
Pierre	Pouget			ICM	Paris		GP2	
Tino	Prell			Department of Neurology, Jena University Hospital, 07747 Jena, Thuringia, Germany<br>Department of Geriatrics, Halle University Hospital, 06120 Halle, Saxony-Anhalt, Germany			ROPAD	
Jack David	Price			Perron Institute	Perth		GP2	
Radha	Procopio			Magna Graecia University	Catanzaro		GP2	
Megan J.	Puckelwartz			Northwestern University	Chicago		GP2	
Cintia Margoth Arm	Puente			Instituto Nacional de Ciencias Neurológicas	Lima		GP2	

Supplemental Online Content: Nonauthor Collaborators
*First name, last name, and suffix (if applicable) are required and will appear in PubMed.

*First Name and Middle Initial(s)	*Last Name	*Suffix (eg, Jr, III)	Academic Degrees	Institution	Location (city, state/province, country)	Role or Contribution, eg, chair, principal investigator	Group (if more than 1 Group listed in the byline) and/or Subgroup (eg, Steering Committee)	
Araliz	Puente			Hospital Ángeles Puebla	Puebla		GP2	
Mark	Pulera			Pharmacology Research Institute, 91316 Encino, California, USA			ROPAD	
Nadya	Pyatigorskaya			Hopital Universitaire Pitie Salpetriere, AP-HP	Paris		GP2	
Andrea	Quattrone			Magna Græcia University of Catanzaro	Catanzaro		GP2	
Joseph	Quinn		MD	Oregon Health & Science University, Portland, OR		Local Site Investigator	PDGENeration	
Nicanor Mori	Quispe			Hospital Nacional Daniel A. Carrión	Lima		GP2	
Amine	Rachdi			Mongi Ben Hamida institute of Neurology			GP2	
Uma	Ragunathan		MS	Navitas Clinical Research, Rockville, MD		Site and Clinical Data Management Core	PDGENeration	
Roopa	Rajan			All India Institute of Medical Sciences	Delhi		GP2	
Edwin Roberto	Ramírez-Benítez			Instituto Nacional de Neurología y Neurocirugía	Mexico City		GP2	
Nomena	Rasaholiarison			Faculty of Medicine, University of Fianarantsoa	Fianarantsoa		GP2	
Anais	Raud			Centre de réadaptation de l'Estuaire & Résidence les jardines de la chenaie	Le Havre		GP2	
Tautanova	Raushan			Astana Medical University	Astana		GP2	
Jason	Raw			Clinical Research Unit, Pennine Acute Hospitals NHS Trust, OL1 2JH Oldham, Greater M			ROPAD	
Ashley	Rawls			University of Florida College of Medi	Gainesville		GP2	
Carlos Matias López	Razquin			H.I.G.A. "Gral. San Martín" - ALAPA	La Plata		GP2	
Raquel	Real			University College London	London		GP2	
Juan Pablo Diaz	Rearte			SIPROSA	Tucumán		GP2	

*First name, last name, and suffix (if applicable) are required and will appear in PubMed.

***First Name and Middle Initial(s)**	***Last Name**	***Suffix (eg, Jr, III)**	Academic Degrees	Institution	Location (city, state/province, country)	Role or Contribution, eg, chair, principal investigator	Group (if more than 1 Group listed in the byline) and/or Subgroup (eg, Steering Committee)	
Katrin	Reetz			Department of Neurology, RWTH Aachen University, 52074 Aachen, North Rhine-Westphalia, Germany JARA-BRAIN Institute Molecular Neuroscience and Neuroimaging, Research Centre Jülich, 52428 Jülich, North Rhine-Westphalia, Germany			ROPAD	
Johnathan	Reiner			Department of Neurology, Movement Disorders Clinic, Rabin Medical Center-Beilinson Hospital, 49100 Petach Tikva, Central District, Israel Sackler Faculty of Medicine, Tel Aviv University, 6997801 Tel Aviv, Tel Aviv District, Israel			ROPAD	
Mariia	Rekaeva			I.K. Akhunbaev Kyrgyz State Medical Academy (KSMA), "Unimed" Clinic	Bishkek		GP2	
Joerg	Rennecke			CENTOGENE GmbH, 18055 Rostock, Mecklenburg-Vorpommern, Germany			ROPAD	
Miguel E.	Rentería			QIMR Berghofer Medical Research Institute	Herston		GP2	
Gonzalo	Revuelta		DO	Medical University of South Carolina, Charleston, SC		Local Site Investigator	PDGENeration	
Alexia T. Sánchez	Reyes			Universidad Fernando Pessoa Canarias	Las Palmas de Gran Canaria		GP2	
Paula	Reyes-Pérez			Universidad Nacional Autónoma de México	Santiago de Querétaro		GP2	
Giulietta	Riboldi		MD	New York University, New York, NY		Local Site Investigator	PDGENeration	
Carlos	Rieder			Federal University of Health Sciences of Porto Alegre	Porto Alegre		GP2	
Ekemini	Riley			Coalition for Aligning Science	Washington		GP2	

*First Name and Middle Initial(s)	*Last Name	*Suffix (eg, Jr, III)	Academic Degrees	Institution	Location (city, state/province, country)	Role or Contribution, eg, chair, principal investigator	Group (if more than 1 Group listed in the byline) and/or Subgroup (eg, Steering Committee)	
Víctor E. Raggio	Risso			Facultad de Medciina, Universidad de la República	Montevideo		GP2	
Beate	Ritz			University of California, Los Angeles	Los Angeles		GP2	
Alejandra Medina	Rivera			Universidad Nacional Autónoma de I	Santiago de Querétaro		GP2	
Mie	Rizig			University College London	London		GP2	
Andrew C	Robinson			The University of Manchester	Manchester		GP2	
Bernardo	Rodrigues		MD, PhD	University of Connecticut, Mansfield, CT		Local Site Investigator	PDGENeration	
Mayela	Rodríguez Violante		MD	Instituto Nacional de Neurología y Neurocirugía, Ciudad de México, México	Mexico City	LATAM Site Investigator	PDGENeration, GP2	
Carlos	Rodriguez-Alarcon			Interlab	Guayaquil		GP2	
Daniel C.	Rohrer			Van Andel Institute	Grand Rapids		GP2	
Federico	Roncaroli			University of Manchester	Manchester		GP2	
Anna De	Rosa			University of Naples Federico II	Naples		GP2	
Raymond	Rosales			Metropolitan Medical Center	Manila		GP2	
Michael	Rose			The Ohio State University Medical Center	Columbus		GP2	
David	Rosenberg			Pharmacology Research Institute, 90720 Los Alamitos, California, USA			ROPAD	
Owen A.	Ross			Mayo Clinic	Jacksonville		GP2	
Simon	Rowe			Neuroscience Research Australia	Sydney		GP2	
Laura Ivete	Rudaks			Concord Repatriation General Hospi	Sydney		GP2	
Dolores Buiza	Rueda			Instituto de Biomedicina de Sevilla	Sevilla		GP2	
Joshua	Ruffner			Parkinson’s Foundation, New York, NY		Core Team	PDGENeration	
Lauren	Ruffrage			University of Alabama at Birmingham	Birmingham		GP2	
Javier	Ruiz Martinez			Department of Neurology, Hospital Universitario Donostia, 20014 San Sebastian, Basqu			ROPAD	
Alejandra E	Ruiz-Contreras			Universidad Nacional Autonoma de I	Mexico City		GP2	
Marta	Ruiz-Lopez			Department of Neurology, University Hospital Cruces, Biocruces Research Institute, 48			ROPAD	
Malia	Rumbaugh			Indiana University, Indianapolis, IN		Genetics Counseling Core	PDGENeration	
Mina	Ryten			University College London	London		GP2	
Fadimatu	Sa'Ad			Federal Teaching Hospital	Gombe		GP2	

Supplemental Online Content: Nonauthor Collaborators
*First name, last name, and suffix (if applicable) are required and will appear in PubMed.

***First Name and Middle Initial(s)**	***Last Name**	***Suffix (eg, Jr, III)**	Academic Degrees	Institution	Location (city, state/province, country)	Role or Contribution, eg, chair, principal investigator	Group (if more than 1 Group listed in the byline) and/or Subgroup (eg, Steering Committee)	
Paula	Saffie Awad		MD, PhD	Clínica Santa María, Santiago, Chile		LATAM Site Investigator	PDGENeration	
Akper	Sagynysh			Multiple Sclerosis and Autoimmune Neurological Disorders Center at the Multidisciplinary City Hospital No. 1	Moscow		GP2	
Zakaria	Saied			National Institute Mongi Ben Hamida of Neurology	Tunis		GP2	
Mohamed	Salama			The American University in Cairo	Cairo		GP2	
Mehri	Salari			Shahid Beheshti University of Medical Science	Tehran		GP2	
Ernestina Castro	Salazar			Unión Medica del Norte	Santiago de los Caballeros		GP2	
Bedia	Samanci			Istanbul University, Faculty of Medicine	Istanbul		GP2	
Sara	Sambin			CIC Neurosciences ,Paris Brain institute	Paris		GP2	
Esther	Sammler			Medical Research Council Protein Phosphorylation and Ubiquitylation Unit, University of Dundee, DD1 5EH Dundee, Dundee City, UK Molecular and Clinical Medicine, Ninewells Hospital and Medical School, University of Dundee, DD1 9SY Dundee, Dundee City, UK			ROPAD	
Rocío Pineda	Sánchez			Instituto de Biomedicina de Sevilla	Sevilla		GP2	
Almudena	Sánchez-Gómez			Hospital Clinic of Barcelona	Barcelona		GP2	
Bruno Lopes	Santos-Lobato			Hospital Ophir Loyola	Belém		GP2	
Arinola	Sanyaolu			University of Lagos	Lagos		GP2	
Gerard	Saranza			Chong Hua Hospital	Cebu		GP2	
Imran	Sarker			National Institute of Neurosciences a	Dhaka		GP2	
Ignacio Juan Keller	Sarmiento			Northwestern University	Evanston		GP2	
Samia Ben	Sassi			Mongi Ben Hmida National Institute of Neurology	Tunis		GP2	

Supplemental Online Content: Nonauthor Collaborators
*First name, last name, and suffix (if applicable) are required and will appear in PubMed.

*First Name and Middle Initial(s)	*Last Name	*Suffix (eg, Jr, III)	Academic Degrees	Institution	Location (city, state/province, country)	Role or Contribution, eg, chair, principal investigator	Group (if more than 1 Group listed in the byline) and/or Subgroup (eg, Steering Committee)	
Batma	Sattarova			Batken Regional Hospital	Batken		GP2	
Rachel	Saunders-Pullman			Department of Neurology, Icahn School of Medicine at Mount Sinai, 10003 New York C			ROPAD, GP2	
Sebastian	Schade			Paracelsus-Elena-Klinik Kassel	Kassel		GP2	
Eva	Schäffer			Kiel University	Kiel		GP2	
Anthony	Schapira			University College London	London		GP2	
Randy	Schekman			University of California, Berkeley	Berkeley		GP2	
Tommaso	Schirinzi			University of Rome Tor Vergata	Rome		GP2	
Ilana	Schlesinger			Rambam Health Care Campus, Technion Faculty of Medicine, 31096 Haifa, Haifa Distric			ROPAD	
Ruth	Schneider		MD	University of Rochester, Rochester, NY		Steering Committee	PDGENeration	
Christine M.	Schofield			Research and Development Unit, Royal Cornwall Hospitals Trust, TR1 3LJ Truro, Cornw			ROPAD	
Anette	Schrag			University College London	London		GP2	
Nils	Schroeter			UKS, University of Saarland	Homburg and Mainz		GP2	
Claudia	Schulte			University of Tübingen	Tübingen		GP2	
Artur F.	Schumacher-Schuh			Neurological Services, Clinical Hospit	Porto Alegre		ROPAD, GP2	
Julie	Schwartzbard		MD	Aventura Neurologists, Aventura, FL		Local Site Investigator	PDGENeration	
Michael	Schwarzschild		MD, PhD	Massachusetts General Hospital, Boston, MA		Steering Committee	PDGENeration	
Burton	Scott			Department of Neurology, Duke University School of Medicine, 27710 Durham, North (			ROPAD	
Laurel	Screven			Global Parkinson's Genetics Program (GP2)	Bethesda		GP2	
Valentin	Selvaggi			Hospital Ramos Mejia	Buenos Aires		GP2	
Smaranda Leu	Semenescu			Hopital Universitaire Pitie Salpetriere, AP-HP	Paris		GP2	
Bimsara Sajotha Na	Senanayake			Institution of Neurology, National Hospital of Sri Lanka	Colombo		GP2	
Gulcin Benbir	Senel			Istanbul University-Cerrahpasa, Cerrahpasa Faculty of Medicine	Istanbul		GP2	
Konstantin	Senkevich			McGill University	Montreal		GP2	
Geidy E.	Serrano			Banner Sun Health Research Institute	Sun City		GP2	
Oiher	Serrano-Asensio			University College London	London		GP2	
Ángel	Sesar			Department of Neurology, University Hospital of Santiago de Compostela, 15706 Santi			ROPAD	
Stuart J.	Shafer			Vero Beach Neurology and Research Institute, 32960 Vero Beach, Florida, USA			ROPAD	

Supplemental Online Content: Nonauthor Collaborators
*First name, last name, and suffix (if applicable) are required and will appear in PubMed.

***First Name and Middle Initial(s)**	***Last Name**	***Suffix (eg, Jr, III)**	Academic Degrees	Institution	Location (city, state/province, country)	Role or Contribution, eg, chair, principal investigator	Group (if more than 1 Group listed in the byline) and/or Subgroup (eg, Steering Committee)	
Anisa	Shahid			Queen Mary University of London	London		GP2	
Ali	Shalash			Ain Shams University Hospital	Cairo		GP2	
Cholpon	Shambetova			Kyrgyz State Medical Academy	Bishkek		GP2	
Ejaz A.	Shamim			Kaiser Permanente, MidAtlantic Perr	Washington		GP2	
Huifang	Shang			West China Hospital Sichuan University	Chengdu		GP2	
Manu	Sharma			University of Tubingen	Tübingen		GP2	
Devin	Sharp			Aligning Science Across Parkinson's	Vancouver		GP2	
Chingiz	Shashkin			International Research Institute of Postgraduate Education	Almaty		GP2	
Chad	Shaw			Baylor College of Medicine	Houston		GP2	
Claire E	Shepherd			Neuroscience Research Australia	Sydney		GP2	
Todd	Sherer			The Michael J Fox Foundation for Parkinson's Research	New York		GP2	
Ray	Sheridan			Geriatric Medicine, Royal Devon and Exeter Hospital NHS Foundation Trust, EX2 5DW E			ROPAD	
Guzel	Shiderova			Institute of Neurology and Neurorehabilitation	Almaty		GP2	
Holly	Shill		MD	Barrow Neurological Institute, Phoenix, AZ	Phoenix	Local Site Investigator	PDGENeration, GP2	
Jung Hwan	Shin			Seoul National University	Seoul		GP2	
Tamara	Shiner			Tel Aviv Sourasky Medical Center	Tel Aviv-Yafo		GP2	
Tomotaka	Shiraishi			Jikei University School of Medicine	Tokyo		GP2	
Lisa	Shulman		MD	University of Maryland, College Park, MD	Baltimore	Local Site Investigator	PDGENeration, GP2	
Bernadette	Siddiqi			The Michael J. Fox Foundation for Parkinson's Research	New York		GP2	
Carolina Candeias d	Silva			Universidade Federal de São Paulo UNIFESP/EPM	São Paulo		GP2	
Monty	Silverdale			Division of Neurology, Salford Royal NHS Foundation Trust, Manchester Academic Health Science Centre, University of Manchester, M6 8HD Manchester, Greater Manchester, UK			ROPAD	

Supplemental Online Content: Nonauthor Collaborators
*First name, last name, and suffix (if applicable) are required and will appear in PubMed.

*First Name and Middle Initial(s)	*Last Name	*Suffix (eg, Jr, III)	Academic Degrees	Institution	Location (city, state/province, country)	Role or Contribution, eg, chair, principal investigator	Group (if more than 1 Group listed in the byline) and/or Subgroup (eg, Steering Committee)	
David	Simon		MD, PhD	Beth Israel Deaconess Medical Center, Boston, MA		Local Site Investigator	PDGENeration	
Cristina	Simonet			Queen Mary University of London	London		GP2	
Tanya	Simuni		MD	Northwestern University, Chicago, IL		Past Steering Committee	PDGENeration	
Lewis M	Singleton			Perron Institute of Neurological and Translational Science	Perth		GP2	
Andrew B	Singleton			Global Parkinson's Genetics Program (GP2)	Bethesda		GP2	
Sue-Faye	Siow			Garvan Institute of Medical Research	Sydney		GP2	
Darshana	Sirisena			CNTH Ragama	Ragama		GP2	
Astros Th.	Skuladottir			deCODE genetics/Amgen Inc., Reykjavik, Iceland Faculty of Medicine, University of Iceland, Reykjavik, Iceland	Reykjavik		GP2	
Laura	Smith			Queen Mary University of London	London		GP2	
Andrew K.	Sobering			Augusta University / University of Georgia Medical Partnership	Augusta		GP2	
Bouchetara Moham	Sofiane			Hospital university of Oran-Algeria	Oran		GP2	
Rodolfo	Solís-Vivanco			Instituto Nacional de Neurología y Neurocirugía	Mexico City		GP2	
J	Solle			The Michael J. Fox Foundation for Parkinson's Research	New York		GP2	
Michael	Sommerauer			University Hospital Bonn	Bonn		GP2	
Lam Hoang	Son			University Medical Center Ho Chi Minh City	Ho Chi Minh City		GP2	
Rani	Sophia			Department of Geriatric Medicine, Yeovil Hospital, BA21 4AT Yeovil, Somerset, UK			ROPAD	
Sophia	Sopromadze			Ivane Javakhishvili Tbilisi State University	Tbilisi		GP2	
Begimai	Sovetbekova			I.K. Akhunbaev Kyrgyz State Medical Academy (KSMA)	Bishkek		GP2	
Cleanthe	Spanaki			University of Crete	Heraklion		GP2	
Mariana	Spitz			Neurology, Pedro Ernesto University Hospital, 20551-030 Rio de Janeiro, Rio de Janeirc			ROPAD	
Konitsiotis	Spyridon			University of Ioannina	Ioannina		GP2	
Eleanor J.	Stafford			University College London	London		GP2	

*First Name and Middle Initial(s)	*Last Name	*Suffix (eg, Jr, III)	Academic Degrees	Institution	Location (city, state/province, country)	Role or Contribution, eg, chair, principal investigator	Group (if more than 1 Group listed in the byline) and/or Subgroup (eg, Steering Committee)	
Julia	Staisch			Ochsner Clinic Foundation	New Orleans		GP2	
Maria	Stamelou			Diagnostic and Therapeutic Centre HYGEIA Hospital	Marousi		GP2	
Pantelis	Stathis			Department of Neurology, Mediterraneo Hospital, 166 75 Glyfada-Athens, Attica, Gree			ROPAD	
Leonidas	Stefanis			Biomedical research Foundation of the Academy of Athens	Athens		GP2	
Kari	Stefansson			deCODE genetics/Amgen Inc., Reykjavik, Iceland Faculty of Medicine, University of Iceland, Reykjavik, Iceland	Reykjavik		GP2	
Hreinn	Stefansson			deCODE genetics/Amgen Inc., Reykjavik, Iceland Faculty of Medicine, University of Iceland, Reykjavik, Iceland	Reykjavik		GP2	
Kathryn	Step			Stellenbosch University	Cape Town		GP2	
Erin Furr	Stimming			The University of Texas Health Science Center at Houston	Houston		GP2	
Fabrizio	Stocchi			University and Institute for Research and Medical Care, IRCCS San Raffaele, 00166 Rom			ROPAD	
Audrey	Strongosky			Mayo Clinic Florida	Jacksonville		GP2	
Carolyn	Sue			Neuroscience Research Australia	Sydney		GP2	
Wenhua	Sun			University of Teubingen	Tübingen		GP2	
Per	Svenningsson			Karolinska Institute	Stockholm		GP2	
Maria	Swanberg			Lund University	Lund		GP2	
Christine	Swanson-Fischer			National Institutes of Health	Rockville		GP2	
Bagzhan	Syzdykova			Astana Medical University	Astana		GP2	
Michele	Tagliati			Department of Neurosurgery, Cedars-Sinai Medical Center, Movement Disorder Progra			ROPAD	
Yen F.	Tai			Division of Medicine and Integrated	London		ROPAD, GP2	
Funmilola	Taiwo			University College Hospital	Ibadan		GP2	
Altynay	Talgatkyzy			Semey Medical University	Semey		GP2	
Manuela	Tan			Oslo University Hospital	Oslo		GP2	
Eng-King	Tan			National Neuroscience Institute	Singapore		GP2	
Ai Huey	Tan			University of Malaya	Kuala Lumpur		GP2	
Beisha	Tang			Central South University	Changsha		GP2	
Ioannis	Tarnanas			Ionian University	Corfu		GP2	
Zaruhi	Tavadyan			Somnus Neurology Clinic	Yerevan		GP2	

***First Name and Middle Initial(s)**	***Last Name**	***Suffix (eg, Jr, III)**	Academic Degrees	Institution	Location (city, state/province, country)	Role or Contribution, eg, chair, principal investigator	Group (if more than 1 Group listed in the byline) and/or Subgroup (eg, Steering Committee)	
Yi Wen	Tay			University of Malaya	Kuala Lumpur		GP2	
Michel	Tchan			Westmead Hospital	Westmead		GP2	
Meron	Teferra			McGill University	Montreal		GP2	
Daniel	Teixeira-dos-Santos			Hospital de Clinicas de Porto Alegre	Porto Alegre		GP2	
Marcela Susana	Tela			Hospital Fernandez	Buenos Aires		GP2	
Saule	Temirbaeva			National Hospital	Bishkek		GP2	
Annelies	Terwecoren			Department of Neurology, Damiaan Hospital, 8400 Ostend, Flanders, Belgium			ROPAD	
Michele	Terzaghi			University of Pavia	Pavia		GP2	
Claudia	Testa		MD, PhD	University of North Carolina - Chapel Hill, Chapel Hill, NC		Local Site Investigator	PDGENeration	
Avner	Thaler			Tel Aviv Sourasky Medical Center	Tel Aviv-Yafo		GP2	
Robert	Thibault			Aligning Science Across Parkinson's	Vancouver		GP2	
Lim Thien	Thien			Island Hospital	Penang		GP2	
Stéphane	Thobois			Hospices civils de Lyon, Hopital Neurologique Pierre Wertheimer	BRON		GP2	
Max	Thom			Parkinson's Foundation, New York, NY		Core Team	PDGENeration	
Sven	Thonke			Department of Neurology, Klinikum Hanau, 63450 Hanau, Hesse, Germany			ROPAD	
Gerd	Tinkhauser			University Hospital Bern	Bern		GP2	
François	Tison			University of Bordeaux, France	Bordeaux		GP2	
Marco	Toffoli			University College London	London		GP2	
Tzi Shin	Toh			University of Malaya	Kuala Lumpur		GP2	
Tessa Du	Toit			UCL	Londo		GP2	
Lars	Tönges			Department of Neurology, St. Josef-Hospital, Ruhr University Bochum, 44791 Bochum, North Rhine-Westphalia, Germany Neurodegeneration Research, Protein Research Unit Ruhr (PURE), Ruhr University Bochum, 44791 Bochum, North Rhine-Westphalia, Germany			ROPAD	
Marta Bonilla	Toribio			Instituto de Biomedicina de Sevilla	Sevilla		GP2	

*First Name and Middle Initial(s)	*Last Name	*Suffix (eg, Jr, III)	Academic Degrees	Institution	Location (city, state/province, country)	Role or Contribution, eg, chair, principal investigator	Group (if more than 1 Group listed in the byline) and/or Subgroup (eg, Steering Committee)	
Santiago Diaz	Torres			Queensland Institute of Medical Research (QIMR Berghofer) / The University of Queensland	Brisbane		GP2	
Giulia	Toschi			Neurology Unit, Neuromotor and Rehabilitation Department, Azienda Unità Sanitaria Locale-IRCCS di Reggio Emilia, 42122 Reggio Emilia, Emilia-Romagna, Italy			ROPAD	
Michelle	Totten			Indiana University, Indianapolis, IN		Genetics Counseling Core	PDGENeration	
Benhassine	Traki			Faculty of Biological Sciences, USTHB Bab Ezzouar, Algiers	Algiers		GP2	
Tai Ngoc	Tran			University Medical Center HCMC	Ho Chi Minh		GP2	
Jaime Fornaguera	Trías			Universidad de Costa Rica	San José		GP2	
Joanne	Trinh			University of Lübeck	Lübeck		GP2	
Bayasgalan	Tserensodnom			Mongolian National University of Medical Sciences	Ulaanbaatar		GP2	
Alexander	Tsiskaridze			Ivane Javakhishvili Tbilisi State University	Tbilisi		GP2	
Khosbayar	Tulgaa			Mongolian National University of Medical Sciences	Ulaanbaatar		GP2	
Vitor	Tumas			Department of Neurosciences and Behavioral Sciences, Ribeirao Preto Medical School of University of São Paulo, 14049-900 São Paulo, São Paulo, Brazil	São Paulo		ROPAD, GP2	
Zhanylsyn	U.Urasheva			West Kazakhstan Marat Ospanov Medical University	Aktobe		GP2	
Oyujin	Ulziibaatar			Mongolian National University of Medical Sciences	Ulaanbaatar		GP2	
Muhammad	Umair			King Abdullah International Medical Research Center	Jeddah		GP2	
Shoaib	Ur-Rehman			University of Science and Technology Bannu	Bannu		GP2	

Supplemental Online Content: Nonauthor Collaborators
*First name, last name, and suffix (if applicable) are required and will appear in PubMed.

*First Name and Middle Initial(s)	*Last Name	*Suffix (eg, Jr, III)	Academic Degrees	Institution	Location (city, state/province, country)	Role or Contribution, eg, chair, principal investigator	Group (if more than 1 Group listed in the byline) and/or Subgroup (eg, Steering Committee)	
Peter Paul	Urban			Department of Neurology, Asklepios Klinik Barmbek, 22307 Hamburg, Hamburg, Germ			ROPAD	
Aigerim	Utegenova			West Kazakhstan Marat Ospanov St	Aktobe		GP2	
Zynan Ruslan	uulu			I.K. Akhunbaev Kyrgyz State Medical Academy (KSMA)	Bishkek		GP2	
Maatali Abdimanap	uulu			Osh State University, Osh City Clinical Hospital	Osh		GP2	
Ha Ngoc Le	Uyen			University Medical Center Ho Chi Minh city	Ho Chi Minh		GP2	
Laura	Vacca			University and Institute for Research and Medical Care, IRCCS San Raffaele, 00166 Rome, Lazio, Italy			ROPAD	
Romain	Valabregue			ICM	Paris		GP2	
Blanca	Valdovinos		MD	University of Rochester, Rochester, NY		Local Site Investigator	PDGENeration	
Enza Maria	Valente			IRCCS Mondino Foundation, Neurogenetics Research Center, 27100 Pavia, Italy Department of Molecular Medicine, University of Pavia, 27100 Pavia, Lombardy, Italy	Pavia		ROPAD, GP2	
Franco	Valzania			Neurology Unit, Neuromotor and Rehabilitation Department, Azienda Unità Sanitaria Locale-IRCCS di Reggio Emilia, 42122 Reggio Emilia, Emilia-Romagna, Italy			ROPAD	
Wim	Vandenberghe			Department of Neurology, University Hospitals Leuven, 3000 Leuven, Flanders, Belgium Department of Neurosciences, KU Leuven, 3000 Leuven, Flanders, Belgium			ROPAD	

Supplemental Online Content: Nonauthor Collaborators
*First name, last name, and suffix (if applicable) are required and will appear in PubMed.

*First Name and Middle Initial(s)	*Last Name	*Suffix (eg, Jr, III)	Academic Degrees	Institution	Location (city, state/province, country)	Role or Contribution, eg, chair, principal investigator	Group (if more than 1 Group listed in the byline) and/or Subgroup (eg, Steering Committee)	
Nora	Vanegas		MD	Baylor College of Medicine, Houston, TX		Local Site Investigator	PDGENeration	
Luciana Rojas	Vazquez			SIPROSA	Tucumán		GP2	
Lydia	Vela-Desojo			Neurology Unit, Hospital Fundación /	Madrid		ROPAD, GP2	
Carlos	Velez-Pardo			Universidad de Antioquia	Medellín		GP2	
Jennifer	Verbrugge		MS, CGC	Indiana University, Indianapolis, IN		Genetics Counseling Core	PDGENeration	
Rossy Cruz	Vicioso			Unión Médica Del Norte, Clínica Universitaria	Santiago de los Caballeros		GP2	
Mari	Vidailhet			Salpêtriere Hospital (AP-HP), Sorbor	Paris		GP2	
Angel	Vinuela			University of Puerto Rico	San Juan		GP2	
Dan	Vitale			Data Tecnica	Bethesda		GP2	
Khang	Vo			University Medical Center	Ho Chi Minh		GP2	
Eva-Juliane	Vollstedt			University of Lübeck	Lübeck		GP2	
Lisa	Voltolina			New York Stem Cell Foundation Reseach Institute	New York		GP2	
Kolawole	Wahab			University of Ilorin	Ilorin		GP2	
Florencia Nicole	Wainberg			Fleni	Buenos Aires		GP2	
Emily	Waldo			Cleveland Clinic	Cleveland		GP2	
Ruth	Walker			James J. Peters Veterans Affairs Medical Center	New York		GP2	
Richard	Walker			Northumbria Healthcare at NHS Fou	Newcastle upon Tyne		GP2	
Lucía	Wang			PARKINSON ARGENTINA/ALAPA	Buenos Aires		GP2	
Thomas	Warner			University College London	London		GP2	
Bart Van De	Warrenburg			Radboud University Medical Center	Nijmegen		GP2	
Sheena	Waters			Queen Mary University of London	London		GP2	
Claire	Wegel			Indiana University	Bloomington		GP2	
Florian	Wegner			Hannover Medical School	Hannover		GP2	
Rimona	Weil			University College London	London		GP2	
Caroline	Weill			Department of Neurology, Faculty of Medicine, Hadassah Medical Organization, Hebrew University, 91120 Jerusalem, Jerusalem District, Israel			ROPAD	

*First name, last name, and suffix (if applicable) are required and will appear in PubMed.

*First Name and Middle Initial(s)	*Last Name	*Suffix (eg, Jr, III)	Academic Degrees	Institution	Location (city, state/province, country)	Role or Contribution, eg, chair, principal investigator	Group (if more than 1 Group listed in the byline) and/or Subgroup (eg, Steering Committee)	
Daniel	Weintraub			U. Pennsylvania	Philadelphia		GP2	
David	Weise			Department of Neurology, Asklepios Fachklinikum Stadtroda, 07646 Stadtroda, Thuringia, Germany<br>Department of Neurology, University of Leipzig, 04103 Leipzig, Saxony, Germany			ROPAD	
Keren Aliza	Weiss			Garvan Institute of Medical Research	Sydney		GP2	
Christoph	Westenberger			University of Lübeck	Lübeck		GP2	
Ana	Westenberger			University of Lübeck	Lübeck		GP2	
Darshana Dias	Wijegunasinghe			Colombo North Teaching hospital	Colombo		GP2	
Ruwani	Wijeyekoon			Association of Sri Lankan Neurologists	Colombo		GP2	
Robert Arthur	Wilcox			Flinders Medical Centre	Bedford Park		GP2	
Nigel	Williams			Cardiff University	Cardiff		GP2	
Erin	Williams			Van Andel Institute	Grand Rapids		GP2	
Caroline	Williams-Gray			University of Cambridge	Cambridge		GP2	
Rebecca	Williamson		MD, PhD	University of Pennsylvania, Philadelphia, PA		Local Site Investigator	PDGENeration	
Jared	Williamson			Kaiser Permanente	Oakland		GP2	
Amanda	Willis			Garvan Institute of Medical Research	Sydney		GP2	
Anne-Marie	Wills		MD	Massachusetts General Hospital, Boston, MA	Boston	Past Steering Committee; Local Site Investigator	PDGENeration, GP2	
Joanne	Wojcieszek			School of Medicine, Indiana University, 46202 Indianapolis, Indiana, USA			ROPAD	
Martin	Wolz			Department of Neurology, Elblandklinikum Meißen, 01662 Meißen, Soxony, Germany			ROPAD	
Katherine	Wong		MD	University of Southern California, Los Angeles, CA		Local Site Investigator	PDGENeration	
Nicholas	Wood			University College London	London		GP2	
Christine	Wools			Epworth hospital	Melbourne		GP2	
Zbigniew K.	Wszolek			Mayo Clinic College of Medicine	Rochester		GP2	

*First name, last name, and suffix (if applicable) are required and will appear in PubMed.

*First Name and Middle Initial(s)	*Last Name	*Suffix (eg, Jr, III)	Academic Degrees	Institution	Location (city, state/province, country)	Role or Contribution, eg, chair, principal investigator	Group (if more than 1 Group listed in the byline) and/or Subgroup (eg, Steering Committee)	
Yihru	Wu			Chang Gung Memorial Hospital	Taoyuan City		GP2	
Ruey-Meei	Wu			National Taiwan University Hospital	Taipei City		GP2	
Lesley Yue	Wu			University College London	London		GP2	
Kathy H. C.	Wu			St Vincent's Hospital Sydney	Darlinghurst		GP2	
Hsiu-Chuan	Wu			Chang Gung Memorial Hospital	Taoyuan City		GP2	
Isabel	Wurster			University of Tübingen	Tübingen		GP2	
Tao	Xie		MD, PhD	University of Chicago, Chicago, IL	Chicago	Local Site Investigator	PDGENeration, GP2	
Georgia	Xiromerisiou			University of Thessaly	Volos		GP2	
Gilad	Yahalom		MD	Shaare Zedek Medical Center, Jerusalem, Israel		Local Site Investigator	PDGENeration, ROPAD	
Addison	Yake			Parkinson's Foundation, New York, NY		Core Team	PDGENeration	
Gul	Yalcin-Cakmakli			Department of Neurology, Faculty of Medicine, Hacettepe University, 06100 Ankara, A			ROPAD	
Tritia	Yamasaki		MD, PhD	University of Kentucky, Lexington, KY		Local Site Investigator	PDGENeration	
Dennis	Yeow			Neuroscience Research Australia	Sydney		GP2	
Aigul. P.	Yermagambetova			West Kazakhstan Marat Ospanov Medical University	Aktobe		GP2	
Rezzak	Yilmaz			University of Ankara	Ankara		GP2	
Mary-Anne	Young			MonoPD	Sydney		GP2	
Cyrus	Zabetian			VA Puget Sound Health Care Systen	Seattle		GP2	
Hengameh	Zahed		MD, PhD	Stanford University, Stanford, CA		Local Site Investigator	PDGENeration	
Anny Coral	Zambrano			Parkinson's Foundation, New York, NY		Core Team	PDGENeration	
Elizabeth	Zauber		MD	Indiana University, Indianapolis, IN		Local Site Investigator	PDGENeration	
Kirsten	Zeuner			Kiel University	Kiel		GP2	
Yared Z.	Zewde			Addis Ababa University	Addis Ababa		GP2	
Gulmira	Zhamilova			Clinical Hospital of the Presidential Administration of the Kyrgyz Republic	Bishkek		GP2	

Supplemental Online Content: Nonauthor Collaborators
*First name, last name, and suffix (if applicable) are required and will appear in PubMed.

***First Name and Middle Initial(s)**	***Last Name**	***Suffix (eg, Jr, III)**	Academic Degrees	Institution	Location (city, state/province, country)	Role or Contribution, eg, chair, principal investigator	Group (if more than 1 Group listed in the byline) and/or Subgroup (eg, Steering Committee)	
Nazira	Zharkinbekova			South Kazakhstan Medical Academy	Shymkent		GP2	
Xiaopu	Zhou			The Hong Kong University of Science and Technology	Kowloon		GP2	
Alexander	Zimprich			Medical University Vienna Austria	Vienna		GP2	
Alexandra	Zirra			Queen Mary University of London	London		GP2	
Simone	Zittel-Dirks			Department of Neurology, University Medical Center Hamburg-Eppendorf, 20246 Ham			ROPAD	
Yair	Zlotnik			Neurology Department, Soroka University Medical Center, 84101 Beer Sheva, Southern			ROPAD	
Rania	Zouari			National institute of neurology mongi ben hmida	Tunis		GP2	
Christiane	Zweier			Inselspital Bern, University of Bern	Bern		GP2	
Rascol				université Toulouse	toulouse		GP2	
Marie-Alexandrine				Hopital Universitaire Pitie Salpetriere, AP-HP	Paris		GP2	
Nathalie	Schell		MD	Insitute of Neurogenetics, Luebeck, (	Lübeck		ROPAD	
Max	Borsche		MD	Insitute of Neurogenetics, Luebeck, (	Lübeck		ROPAD	
Henrike	Hanssen		MD	Insitute of Neurogenetics, Luebeck, (	Lübeck		ROPAD	
Tatjana	Usnich		MD	Insitute of Neurogenetics, Luebeck, (	Lübeck		ROPAD	
