# Supplementary material for: Genetic Testing by Age at Onset in Parkinson Disease
Source: JAMA Neurol. 2026 May 11;83(7):711–3. doi: 10.1001/jamaneurol.2026.1112 (PMC13162140; doi:10.1001/jamaneurol.2026.1112)
Supplement: Supplement 3. — Data sharing statement [file jamaneurol-e261112-s003.pdf]

Data Sharing Statement

Balck. Genetic Testing by Age at Onset in Parkinson Disease. *JAMA Neurol*. Published May 11, 2026. doi:10.1001/jamaneurol.2026.1112

Data
Data available: Yes
Data types: Other (please specify)
Additional Information: Data used in the preparation of this article were obtained, among others, from the Global Parkinson's Genetics Program (GP2; https://gp2.org). Specifically, we used Tier 2 data from GP2 release 9 (DOI 10.5281/zenodo.14510099). GP2 data are available on AMP PD (https://amp-pd.org). Data were also obtained from ROPAD, PD GENEration, the MDSGene database, and two German observational studies, these datasets can be accessed by individual request to the corresponding author. All code generated for this article, and the identifiers for all software programs and packages used, are available on GitHub (https://github.com/GP2code/Genetic_Testing_in_PD) and were given a persistent identifier via Zenodo (DOI: 10.5281/zenodo.17567723).
How to access data: see above
When available: With publication

Supporting Documents
Document types: Statistical/analytic code
How to access documents: see above
When available: With publication

Additional Information
Who can access the data: see above
Types of analyses: see above
Mechanisms of data availability: see above
